# Autophagy in Alcohol-Induced Multiorgan Injury: Mechanisms and Potential Therapeutic Targets

**DOI:** 10.1155/2014/498491

**Published:** 2014-07-17

**Authors:** Yuan Li, Shaogui Wang, Hong-Min Ni, Heqing Huang, Wen-Xing Ding

**Affiliations:** ^1^Department of Pharmacology, Toxicology and Therapeutics, The University of Kansas Medical Center, MS 1018 3901 Rainbow Boulevard, Kansas City, KS 66160, USA; ^2^Laboratory of Pharmacology and Toxicology, School of Pharmaceutical Sciences, Sun Yat-Sen University, Guangzhou 510006, China

## Abstract

Autophagy is a genetically programmed, evolutionarily conserved intracellular degradation pathway involved in the trafficking of long-lived proteins and cellular organelles to the lysosome for degradation to maintain cellular homeostasis. Alcohol consumption leads to injury in various tissues and organs including liver, pancreas, heart, brain, and muscle. Emerging evidence suggests that autophagy is involved in alcohol-induced tissue injury. Autophagy serves as a cellular protective mechanism against alcohol-induced tissue injury in most tissues but could be detrimental in heart and muscle. This review summarizes current knowledge about the role of autophagy in alcohol-induced injury in different tissues/organs and its potential molecular mechanisms as well as possible therapeutic targets based on modulation of autophagy.

## 1. Introduction

Chronic or acute alcohol abuse often leads to liver injury associated with alcoholic hepatitis, liver fibrosis, cirrhosis, and liver cancer [1]. In addition to the liver, alcohol abuse also induces a variety of other tissue injuries including pancreatitis [2, 3], cardiomyopathy [4, 5], neurotoxicity [6], muscle loss [7], impaired immune functions [1], endocrine and fetal abnormalities [8], and osteoporosis [9]. According to the National Institute on Alcohol Abuse and Alcoholism (NIAAA), more than 18 million adults are affected by alcoholism in the United States, which costs 27 billion dollars for treating alcohol-attributable diseases.

The mechanisms for alcohol-induced detrimental effects in various tissues/organs have been extensively studied, which involves genetic and environmental factors as well as altering multiple cellular signaling pathways. These mechanisms involve ethanol and its metabolites that induce reactive oxygen species (ROS) generation, lipid peroxidation, cytokine expression/inflammation, organelle damage, and stress and activate both apoptotic and necrotic cell death pathways [10]. However, the full picture especially the cellular adaptive and protective mechanisms against ethanol-induced stress and tissue injury has not been well depicted yet. Currently, the treatment for chronic alcohol diseases is not very effective owing largely to our incomplete understanding of the cellular adaptive response to ethanol toxicity.

Macroautophagy (hereafter referred to as autophagy) is a genetically programmed, evolutionarily conserved intracellular degradation pathway in response to stress. It is involved in the trafficking of long-lived proteins and cellular organelles to the lysosome for degradation to maintain cellular homeostasis. It is tightly controlled by over 30 autophagy-related (Atg) genes [11]. Autophagy is generally considered as a cell survival mechanism in response to various stress conditions and plays a critical role in human physiology and diseases [12].

Accumulating evidence has shown that altered autophagy is implicated in the pathogenesis and protection of ethanol-induced tissue injury [13–15]. This review aims to summarize current knowledge about the role of autophagy in alcohol-induced injury in multiple tissues/organs and their underlying molecular mechanisms as well as potential therapeutic targets based on modulation of autophagy.

## 2. Ethanol Metabolism and Its Effect on Tissue Injury

Ethanol is metabolized through several pathways. Predominantly alcohol is metabolized by alcohol dehydrogenase (ADH) into acetaldehyde, a highly reactive byproduct, and acetaldehyde is further metabolized by aldehyde dehydrogenase (ALDH) into acetate, a more harmless substance. The most important two isoforms of ALDH are the cytosolic ALDH1 and the mitochondrial ALDH2 [16]. It has been reported that there are functional polymorphisms among ADH and ALDH that can influence the susceptibility of humans to alcoholism [17]. The oxidation of ethanol mainly not only occurs in liver [18], but also occurs in pancreas [19], heart [5], and other organs [20], accompanied by conversion of nicotinamide adenine dinucleotide (NAD^+^) into its reduced form, NADH, which plays critical roles in cellular redox status. Besides, ethanol could also be metabolized by cytochrome P450 family 2, subfamily E, polypeptide 1 (Cyp2E1), and catalase. Excessive ethanol exposure largely induces Cyp2E1, which not only mediates and activates reactions of many toxicological substrates, but also generates ROS leading to cellular damage [21]. Additionally, a minimal amount of ethanol can also be metabolized via two nonoxidative pathways. In the first pathway, ethanol interacts with fatty acid and generates fatty acid ethyl ester (FAEE), which is catalyzed by FAEE synthase in many tissues and organs [22]. FAEE was thought to have minor effect and mainly considered as a diagnostic marker but accumulated evidence shows that FAEE exacerbates injury after ethanol exposure especially in pancreas [23, 24], liver [25], and heart [23, 25, 26] and is facilitated by ADH deficiency [27]. One explanation of cytotoxicity of FAEE is that FAEE binds to mitochondria membrane and with its hydrolysis products, fatty acids, causes damage by uncoupling oxidative phosphorylation [28]. In the second pathway, phospholipase D (PLD), which normally breaks down phospholipids to generate phosphatidic acid (PA), reacts with ethanol to generate phosphatidyl ethanol. Following chronic consumption of large amounts of alcohol, phosphatidyl ethanol may accumulate to detectable levels because it is poorly metabolized. However, the effects of phosphatidyl ethanol on cellular functions remain to be further studied [20].

Alcohol is easily absorbed and could be metabolized and impact almost all over the body. Matured brain is less affected compared with developing brain due to the blood-brain barrier, but excessive oxidative stress and intracellular Ca(2+) release induced by ethanol could impair the barrier function [29]. Dysregulated metabolites lead to changes in carbohydrate metabolism, cell death signaling, mitochondria damage, and epigenetic regulation [30]. Apoptosis and necrosis induced or exacerbated by alcohol metabolism have been studied in liver [31, 32], pancreas [33, 34], heart, brain [35, 36], developing brain [37], and skeletal muscle [38]. The important mechanisms that are thought to be involved in alcohol-induced liver injury include (1) oxidative stress and lipid peroxidation [39, 40]; (2) liver hypoxia [41, 42]; (3) endoplasmic reticulum (ER) stress and activation of mitogen activated protein kinases (MAPK) [43, 44]; and (4) inhibition of proteasome and lysosomal functions that causes hepatomegaly and Mallory inclusion bodies [45–47]. Recent evidence has also suggested that chronic alcohol consumption induces necroptosis in mouse livers, which is dependent on the receptor interacting protein 3 (RIP 3) [48]. The effect of ethanol metabolites in different tissue/organ autophagy and cell injury will be further discussed below.

## 3. Role of Autophagy in Alcoholic Liver Disease

Liver is one of the most active organs, which plays a central role in regulating the overall organism energy balance by controlling carbohydrate and lipid metabolism. Under physiological conditions, liver also serves as a major buffering system to ensure other tissues to function normally by maintaining the homeostasis of macro- and micronutrient. To accomplish the vital missions, liver may rely on autophagy, the cellular catabolic process to breakdown macromolecules, lipids, and damaged/excess organelles. Indeed, liver-specific autophagy gene knockout (KO) mice have disrupted metabolism of proteins, glucose, and lipids, accumulated damaged and excess organelles such as mitochondria and peroxisomes, and resulted in increased cell death, inflammation, and liver tumorigenesis [49–52]. Alcoholic liver disease (ALD) also involves the disruption of cellular metabolism of proteins and lipids and homeostasis of organelles such as mitochondria and endoplasmic reticulum (ER) resulting in increased cell death that contributes to alcoholic hepatitis, liver fibrosis, cirrhosis, and liver cancer [1, 53]. All these pathogenic events are intimately related to the autophagic process, and modulating autophagy may thus affect the ALD pathogenesis. Indeed, accumulating evidence now indicates that autophagy plays a critical role in the pathogenesis of ALD [13–15].

In the past several years, many animal models as well as* in vitro* cell culture models have been developed to study ALD. While baboons, pigs, and rats have been used to study ALD, mice have been predominantly used in current ALD research. The animal models for ALD include acute alcohol gavage,* ad libitum* oral alcohol in drinking water, intragastric infusion (Tsukamoto-French model), chronic Lieber-DeCarli diet ethanol feeding, and the most recent Gao-binge (chronic + binge) models [53, 54]. In addition to* in vivo* models, primary cultured hepatocytes and engineered HepG2 cells that are stably expressing Cyp2E1 and ADH are also used to study alcohol-induced pathophysiological changes in hepatocytes [55, 56]. Unfortunately, all these current models can only capitulate some of the phenotypes of ALD, which do not progress beyond liver steatosis, inflammation, and injury. Owing to the complexity of the models to study ALD, lack of reliable markers, and the difficulty to monitor autophagic flux in chronic ALD models, controversial results regarding the autophagy status (activated or impaired) during the pathogenesis of ALD have been reported. Some possible explanations for these contradictory results will be discussed in detail below.

Autophagy was first described by de Duve and Wattiaux in the rat liver that was challenged with glucagon in the 1960s [57], but the molecular characterization of autophagy began in the 1990s by Ohsumi and Wolf's laboratories using yeast genetic screens that led to the discovery of a group of essential Atg genes in yeast [58, 59]. These Atg genes were later found to be highly conserved in mammals. Autophagy is a highly dynamic process involving several key steps: (1) the first step is to activate the preinitiation complex that is composed of Unc-51 like kinase 1- (ULK1-) FAK family-interacting protein of 200 kDa (FIP200)-Atg13, which is negatively regulated by the upstream nutrient sensor the mammalian target of rapamycin complex 1 (mTORC1) and positively regulated by the energy sensor Amp-activated protein kinase (AMPK), leading to the initiation of autophagosome biogenesis [60–62]; (2) the preinitiation complex and the ER-resident SNARE protein syntaxin 17 (STX17) then recruit Atg14L to the rough ER or ER-mitochondria contact site, which further recruits Beclin-1 and VPS34 to the autophagosome initiation site on the rough ER [63, 64]. VPS34 then promotes the generation of phosphatidylinositol 3-phosphate- (PI3-P-) enriched autophagosome initiation sites that further recruit PI3-P effectors including double FYVE domain-containing protein 1 (DFCP1), WD-repeat interacting protein with phosphoinosides 1 (WIPI1), and WIPI2 to initiate the biogenesis of autophagosomes [65–67]. This complex is positively regulated by activating molecule in Beclin-1-regulated autophagy (Ambra-1) [68], UV irradiation resistance-associated gene (UVRAG) [69], Bif-1/Endophilin B1 [70], and AMPK and negatively regulated by binding to Bcl-2 [71], Bcl-xL, run domain protein as Beclin-1 interacting and cysteine-rich containing (Rubicon) [72, 73], AKT, and epidermal growth factor receptor (EGFR); (3) next, two ubiquitin-like conjugation systems, Atg7-Atg3-microtubule-associated light chain (LC3) and the Atg12-Atg5-Atg16L1 complex, regulate conjugation of phosphatidylethanolamine with LC3 (called LC3-II), which expands the autophagosome membrane [74–76]. Atg9 also delivers membranes from* trans*-Golgi network/endosomes to the site of autophagosome biogenesis in an ULK1- and VPS34-dependent manner to promote the expansion of the autophagosome membrane [77]. The Atg12-Atg5-Atg16L complex only transiently attaches to the autophagosomal membranes and is later dissociated from the autophagosomal membranes [78], and PI3-P is also dephosphorylated locally by the phosphatases myotubularin-related protein 3 (MTMR3, also called Jumpy) upon closure of the autophagosomes [79, 80]; (4) finally, autophagosomes fuse with lysosomes/endosomes to form autolysosomes, which is mediated by Rab7, Lamp1/2, and the SNARE protein STX17 [81–83]. After fusion, the outer membrane of LC3-II is dissociated from the autolysosomal membrane through a deconjugation process mediated by Atg4B, and inner membrane LC3-II is degraded together with autophagosome cargos [84, 85].

Due to the complex dynamic nature of the autophagic process, it is very challenging to monitor autophagy in a quantitative way, in particular* in vivo* in whole animal tissues/organs. While LC3-II is widely used to monitor the autophagic process, LC3-II itself is also degraded in the autolysosomes. Thus an autophagic flux assay, which monitors LC3-II levels with or without a lysosomal inhibitor such as chloroquine or bafilomycin A1, has been recommended to determine autophagy status by the autophagy research community [86]. In addition, the level of p62/sequestosome 1 (SQSTM1) has also been suggested to use another marker for autophagic flux because p62/SQSTM1 is normally degraded in response to starvation and accumulated in genetic autophagy gene deleted mouse livers [50, 87]. However, the levels of p62/SQSTM1 may not always be suitable to monitor autophagic flux because its levels are also regulated at the transcriptional level, which is often induced in many experimental autophagy models including prolonged starvation conditions [88]. As discussed above, there are some controversial reports regarding the autophagy status in ALD research. The reasons behind these controversial reports are likely due to the complexity of autophagy assays and the use of many different ALD models. It is generally agreed that acute ethanol (binge) treatment increases autophagy in mouse livers and in primary cultured murine hepatocytes, a conclusion that is supported by autophagic flux data in these studies [89, 90]. In contrast, impairment of autophagy has also been reported in ethanol-treated Cyp2E1 overexpressing HepG2 cells or in Cyp2E1 knock-in mice that were given acute alcohol twice a day for four days [91, 92]. However, only a decreased LC3-II/I ratio was observed and no autophagic flux assays were conducted in these studies. Using HepG2 cells that stably overexpressed both Cyp2E1 and ADH, Thomes et al. [93] reported that ethanol treatment not only increased autophagosome synthesis but also impaired lysosomal degradation. However, the autophagic flux data in this study actually supported an increase in autophagy [93]. Early works from the same group showed that ethanol treatment may increase lysosomal pH and impair cathepsin maturation [94, 95]. Thus it is possible that increased autophagy by ethanol treatment may serve as a compensatory mechanism in response to ethanol-induced mild impaired lysosomal functions. In contrast to acute alcohol treatment, it has been generally thought that chronic alcohol consumption may have impaired autophagy in the liver because it has long been shown that chronic alcohol administration leads to hepatomegaly and protein accumulation in liver [96–98]. In a recent chronic ethanol feeding study, Lin et al. performed autophagic flux studies in mice that were fed an ethanol diet for 4 weeks and increased autophagic flux was found in that study [99]. However, we should interpret these data cautiously since the autophagic flux assay was only performed at one time point after the 4-week feeding, while ideally, autophagic flux assays should be applied to multiple time points owing to the dynamic nature of autophagy. In addition, the autophagy status in other ALD models, such as the Tsukamoto-French model and Gao-binge model, has not been reported. Moreover, in addition to hepatocytes, whether and how ethanol would affect autophagy on other liver cells, such as hepatic stellate and Kupffer cells, and their impacts on ALD are largely unknown. Regardless of the controversies on autophagy status, it has been unanimously shown that activating autophagy is beneficial against ALD in various ALD models [89, 90, 99, 100].

## 4. Possible Mechanisms Affecting the Autophagy Process Induced by Alcohol

As discussed above, autophagy is a dynamic multistep process that is tightly regulated by many signaling pathways involving nutrients, energy, and stress response. Below we discuss the regulating pathways of autophagy that have been shown to be affected by alcohol.

### 4.1. Class I PI3K-Akt-mTOR

As discussed above, mTORC1 is a negative regulator at the preinitiation complex to regulate the initiation of autophagosome biogenesis [101]. mTOR is part of two structurally and functionally different complexes, mTORC1 and mTORC2. The former complex is sensitive to rapamycin and plays a major role in regulation of cell growth and autophagy. mTORC1 is also a sensor of various signals including growth factors, insulin, nutrients, energy status, and cellular stressors. In nutrient-sufficient condition, growth factors activate the class I phosphoinositol-3-kinase (PI3K) to catalyze PIP3 and subsequently activate Akt, which then further activates downstream mTORC1 and inhibits autophagy. mTORC2 may also negatively regulate autophagy because it is required for full activation of Akt [102, 103]. Suppression of Akt and mTOR are common mechanisms of autophagy induction, which is also affected by ethanol treatment. It has been demonstrated that acute ethanol-treated mouse liver and chronic ethanol-treated rat liver had increased expression of phosphatase and tensin homolog (PTEN) resulting in the suppression of Akt [104, 105]. We also found that acute ethanol treatment decreased the level of phosphorylated Akt in mouse liver [89]. Besides, ethanol treatment also inhibited mTORC1 activity in primary cultured mouse hepatocytes [90]. More importantly, pharmacological inhibition of mTOR by either rapamycin or Torin 1 significantly suppressed acute ethanol-induced liver steatosis and injury [15, 90]. More future studies are needed to further determine the detailed time-course changes of mTOR and their associations with autophagy during chronic alcohol feeding.

PI3K/Akt activation is known to enhance sterol regulatory element-binding protein-1 (SREBP-1) [106]. In addition, a system biology-based integrative computational analysis also suggests that SREBP-1 may coordinate autophagy-lysosomal activities and lipid metabolism [107]. Interestingly, application of wortmannin, a PI3K/Akt inhibitor, showed dual effects on acute ethanol-induced fatty liver depending on dose [108]. Low dose wortmannin inhibited whereas high dose of wortmannin exacerbated acute ethanol-induced steatosis in mouse livers. It has been suggested by the authors that high dose of wortmannin might inhibit hepatic autophagy whereas low dose of wortmannin alleviated the rise of hepatic triglycerol possibly by inhibiting SREBP-1 via PI3K/Akt inhibition [108]. These findings suggest that special attention should be paid to the use of PI3K/Akt inhibitor in alcohol-induced fatty liver studies.

### 4.2. AMPK

AMPK is a key energy sensor that regulates cellular metabolism and energy homeostasis. AMPK can directly inhibit mTOR through increased phosphorylation of TSC2 [109, 110] and Raptor [111], which activates autophagy. AMPK also promotes autophagy by phosphorylating ULK1 [109, 112], VPS34, and Beclin-1 [113]. However, it has been shown that administration of an AMPK activator adenosine, 5-amino-4-imidazole carboxamide riboside (AICAR), suppresses autophagy in hepatocytes [114]. Moreover, administration of compound C, an AMPK inhibitor, activates autophagy via AMPK-independent blockade of the Akt/mTOR pathway, which overcomes the expected inhibitory effect on autophagy via AMPK inhibition in cancer cells [115]. Several lines of evidence show that AMPK activity is reduced in liver by ethanol consumption, which is believed to promote fatty liver through activation of SREBP-1 and upregulation of lipin-1 expression [116, 117]. The exact role of AMPK in ethanol-induced autophagy is not clear but it is possible that ethanol induces autophagy independent of AMPK activation.

### 4.3. ADH, Cyp2E1, and ROS

In liver, ethanol is mainly metabolized by ADH and Cyp2E1, which promotes the generation of ROS and other reactive toxic metabolites. Interestingly, ethanol-induced autophagy requires its metabolism and ROS production because autophagic flux was only induced in HepG2 cells stably expressing ADH and Cyp2E1 but not in parental HepG2 cells [90, 93]. Moreover, blocking ADH and Cyp2E1 by 4-methylpyrazole or inhibiting ROS by antioxidants also reversed the inhibition of mTOR and diminished increased GFP-LC3 puncta [90, 93]. It seems that alcohol oxidation by Cyp2E1 is also important for alcohol-induced inhibition of cellular proteasome activity and increased autophagosome numbers [118]. It has been proposed that ROS may activate autophagy through modulating the oxidization of Atg4, an autophagy machinery protein important for generating and recycling of LC3-II [119]. As for the importance of Cyp2E1-mediated ethanol metabolism on ethanol-induced autophagy changes, several studies have reported that ethanol-treated HepG2 cells that are overexpressing Cyp2E1 and ethanol-treated* Cyp2E1* KO or knock-in mice showed decreased LC3-II levels [91, 92, 100, 120]. While the authors concluded that Cyp2E1-mediated metabolism of ethanol may lead to inhibition of autophagy in these studies, autophagic flux assays were not conducted in these studies. Future studies are needed to further confirm the autophagy status in ethanol-treated* Cyp2E1* KO or knock-in mice by performing an autophagic flux assay.

### 4.4. FoxO3 and SIRT1

Forkhead box-containing protein class O (FoxO) family of DAF-16 like transcription factors are evolutionarily conserved transcriptional factors that regulate the expression of genes involved in multiple cellular functions including oxidative stress, glucose metabolism, apoptosis, cell cycle transition, and DNA repair [121, 122]. Four FoxO proteins including FoxO1, FoxO3, FoxO4, and FoxO6 are found in mammals, which have redundant yet distinctive roles in regulating gene expression. While FoxO1, FoxO3, and FoxO4 are ubiquitously expressed in most tissues, FoxO6 is mainly expressed in neurons. Studies from gene KO mice show that* FoxO1* KO mice are embryonically lethal due to impaired angiogenesis but* FoxO4* KO mice are viable with decreased migration of vascular smooth muscle cells [123]. While* FoxO3* KO mice are also viable, female mice are infertile due to ovarian activation and they also have spontaneous T cell activation and lymphoproliferation with time [124]. FoxO3 mainly regulates the expression of genes responsible for oxidative stress, apoptosis, cell cycle transition, and DNA repair but FoxO1 is more important in regulating glucose and lipid metabolism. All the FoxO family proteins are subjected to multiple posttranslational modifications, including phosphorylation, acetylation, methylation, and ubiquitination [122]. Akt-mediated phosphorylation of FoxO3 causes its nuclear exclusion and thus inactivates FoxO3. It seems that the acetylation of FoxO3 mainly regulates the specificity of a subset of FoxO3 target genes by increasing the expression of antioxidant genes and suppressing the expression of apoptosis genes in response to oxidative stress [125], whereas methylation of FoxO3 at K270 results in loss of DNA binding [126].

Increasing evidence now suggests that FoxO family proteins can also regulate autophagy by three distinctive mechanisms: direct transcriptional regulation of Atg gene expression [127, 128], transcriptional regulation of glutamine synthetase expression and increasing intracellular glutamine levels [129], and interaction of cytosolic FoxO1 with Atg7 independent of its transcription activity [130].

Sirtuin 1 (Sirt1) belongs to the evolutionarily conserved sirtuin family, which are NAD-dependent class III protein deacetylases. There are 7 sirtuins (Sirt1–7) that have been identified in mammals, which have distinct cellular locations. Sirt1, 6, and 7 are mainly in the nucleus, Sirt2 is mainly in the cytosol, and Sirt3, 4, and 5 are found in the mitochondria [131, 132]. Sirt1 has a broad range of physiological functions including the control of aging, metabolism, and gene expression by promoting the deacetylation of a variety of substrates from histones to nonhistone proteins. Increasing evidence suggests that sirtuins also play roles in regulating autophagy.* Sirt1* KO mouse embryonic fibroblasts have decreased autophagy in response to starvation, which is accompanied by increased acetylated Atg5, Atg7, and LC3 proteins, although it remains unclear how increased acetylation of these proteins affects their functions on autophagy [133]. In response to stress, cytosolic FoxO1 is dissociated from Sirt2 resulting in an increase of acetylated FoxO1. Acetylated FoxO1 then binds to Atg7 and promotes autophagy in some human cancer cells [130]. Adult-onset and long-term calorie restriction in mice increased Sirt1 expression in aged kidney and attenuated hypoxia-associated mitochondrial and renal damage by enhancing Bcl2/adenovirus E1B 19-kDa interacting protein 3- (Bnip3-) dependent autophagy. This increased autophagy was found to be regulated by Sirt1-mediated FoxO3 deacetylation resulting in increased expression of Bnip3 under hypoxia conditions [134].

We recently demonstrated that acute ethanol treatment increased the expression of Atg genes in mouse liver and in primary cultured mouse and human hepatocytes, which was accompanied by increased hepatic nuclear accumulation of FoxO3 [89]. Acute ethanol treatment decreased the level of phosphorylated Akt, causing decreased FoxO3 phosphorylation at Ser253, which could account for increased nuclear FoxO3. Resveratrol increases Sirt1 activity by promoting its binding with both NAD^+^ and the acetylated substrate through allosteric interaction [135]. Indeed, we found that activation of Sirt1 by resveratrol increased deacetylation of FoxO3 and enhanced ethanol-induced expression of Atg genes [89]. Moreover, we found that* FoxO3* KO mice had decreased expression of Atg genes and had increased steatosis and liver injury compared to wild type mice after acute ethanol treatment [89]. These findings indicate that FoxO3-mediated autophagy plays a protective role against alcohol-induced steatosis and liver injury. It has been suggested that ethanol consumption may inhibit Sirt1 via increased NADH/NAD^+^ ratio through its metabolism. This may lead to the inhibition of autophagy in the liver either through FoxO3-dependent or independent mechanisms. However, ethanol consumption can also inhibit Akt phosphorylation, which can lead to increased nuclear retention of FoxO3 and ultimately increased expression of autophagy genes. Thus it is possible that Akt-mediated FoxO3 nuclear retention would be more important or dominant in regulating the expression of Atg genes than Sirt1-mediated acetylation of FoxO3. Nevertheless, our results suggest that activation of Sirt1, such as by using resveratrol, can further enhance ethanol-induced FoxO3-mediated expression of Atg genes. More studies are definitely needed to determine whether other FoxO and sirtuin family proteins are also involved in autophagy in alcohol-induced liver injury.

Nepal and Park recently also reported a link between AMPK/FoxO3 and autophagy when they studied the protective effect of globular adiponectin (gAcrp) on ethanol-treated HepG2 cells [136, 137]. The fat-derived hormone adiponectin is known to be protective in ALD [138]. Greer et al. showed that gAcrp restored ethanol-induced suppression of autophagy genes including* Atg5* and autophagosome formation, which was accompanied by FoxO3 translocation in HepG2 cells. Silencing FoxO3 or its upstream regulator AMPK [139] abrogated the restoration, indicating the importance of FoxO3 and AMPK in ethanol-mediated expression of autophagy genes. However, since HepG2 cells are human hepatoma cells and can barely metabolize ethanol, the interpretation of these results needs to be cautious.

### 4.5. Methionine, SAM, and Methylation

Methionine is a sulfur-containing essential amino acid that is important in biogenesis of cysteine, carnitine, taurine, lecithin, phosphatidylcholine, and other phospholipids. Catalyzed by methionine adenosyltransferase, a liver-specific enzyme, methionine is metabolized into S-adenosylmethionine (SAM), which is a universal methyl donor. After transferring the methyl group, SAM becomes S-adenosylhomocysteine, which can be further converted to adenosine and homocysteine via S-adenosylhomocysteine hydrolase. Homocysteine is a source for generation of methionine through methionine synthase (MS) and glutathione through cystathionine b-synthase [140]. Aberrant methionine metabolism has been well documented in ALD [140–142]. Ethanol exposure inhibits MS activity resulting in decreased hepatic methionine levels [140]. Cells activate a compensatory pathway in methionine metabolism by increasing betaine homocysteine methyltransferase activity, but this pathway is compromised under extended chronic alcohol exposure resulting in a general decrease of hepatic SAM and essential methylation reactions [141].

**Figure 1:**
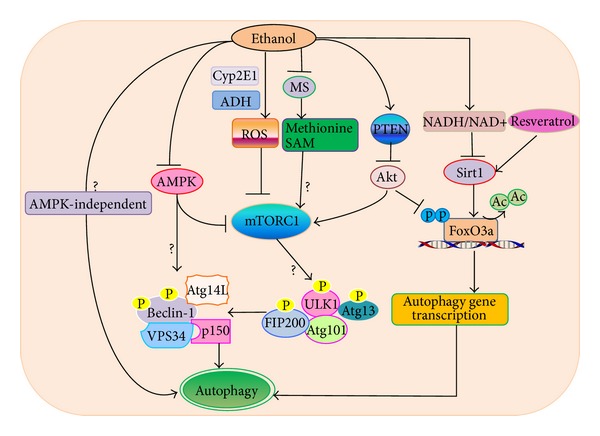
The major molecular pathways and targets in alcohol-induced autophagy changes in hepatocytes. Ethanol modulates autophagy through multiple mechanisms. (1) Ethanol-induced autophagy requires ethanol metabolism and ROS production. ROS may activate autophagy by further suppressing mTOR. (2) Alcohol (ethanol) consumption inhibits methionine synthase (MS) resulting in decreased methionine and S-adenosylmethionine (SAM) levels. Methionine and SAM inhibit autophagy by activating mTORC1. Thus it is possible that ethanol-induced decreased methionine and SAM will inhibit mTORC1 resulting in autophagy activation although this has not been directly tested in the alcohol model (?). (3) Ethanol may also suppress Akt through the upregulation of PTEN and in turn inhibits mTORC1 to induce autophagy. (4) Ethanol-induced impaired AMPK and Akt may counteract each other on mTOR, and impaired Akt plays a dominant role toward the inhibition of mTOR. (5) Decreased Akt can also trigger autophagy through the activation of FoxO3 by promoting the dephosphorylation and nuclear retention of FoxO3. Increased NADH/NAD^+^ ratio through ethanol metabolism inhibits Sirt1 activity resulting in increased acetylated FoxO3. Increased acetylated FoxO3 may decrease FoxO3-mediated expression of autophagy genes, which can be abolished by resveratrol that activates Sirt1. (6) Other AMPK-independent pathways remain to be determined in alcohol-induced autophagy (?). (7) mTORC1 negatively regulates autophagy through direct phosphorylation of ULK1 to inactivate ULK1 complex activity. ULK1 directly phosphorylates Beclin-1 and enhances VPS34 kinase activity to promote autophagy. AMPK positively regulates autophagy by suppressing mTORC1 activity through phosphorylation of TSC2 and raptor and by promoting VPS34 kinase activity through phosphorylation of Beclin-1. Activated VPS34 increases the production of phosphatidylinositol 3-phosphate (PI3P), which promotes the biogenesis of autophagosomes although the activities of ULK1 and VPS34 after alcohol exposure still remain to be determined (?).

Emerging evidence shows that posttranslational modification of proteins with methylation may play important roles in regulating autophagy in at least three aspects: methylation of protein phosphatase 2A (PP2A) to negatively regulate autophagy through modulating target of rapamycin (TOR) [143], epigenetic regulation of autophagy gene transcription through the methyltransferase G9a [144], and arginine methylation in selective autophagy [145]. In a recent yeast study, it was found that methionine and SAM inhibited autophagy and promoted growth through the protein phosphatase methyltransferase 1 (Ppm1p), which increases PP2A methylation. Methylated PP2A promoted the dephosphorylation of natriuretic peptide receptor B (Npr2), a yeast phosphoprotein that negatively regulates TORC1, resulting in TORC1 activation and autophagy inhibition [143]. However, whether methionine and SAM would also inhibit autophagy in mammals through similar mechanisms remains to be studied. The H3K9 methyltransferase G9a was also reported to inhibit autophagy by inducing an increase of dimethylated H3K9 (H3Kme2), which repressed the expression of several essential Atg genes including LC3B, WIPI1, and diabetes and obesity-regulated (DOR). Upon autophagy induction, G9a leaves the promoter region of* LC3B* to release its repression on the expression of* LC3B* and other Atg genes to promote autophagy [144]. For selective autophagy, it is known that the autophagy receptor complex is important for mediating recognition of cargos (such as ubiquitinated mitochondria) and also binds with autophagy machinery proteins (such as LC3), which allows the cargo to be selectively removed [146–149]. Phosphorylation of p62/SQSTM1 and Atg32, two important autophagy receptor proteins, has been shown to play important roles in selective removal of protein aggregates and mitochondria [150–152]. Optineurin, another autophagy receptor protein, is also phosphorylated by the protein kinase TBK1, which enhances its binding with LC3 resulting in selective autophagic clearance of cytosolic* Salmonella enterica* [153]. In addition to phosphorylation, arginine methylation is another major type of protein posttranslational modification and is catalyzed by protein arginine methyltransferases (PRMT). Interestingly, a recent study showed that mutations in* C. elegans epg-11*, a homologue of mammalian* PRMT1*, led to the defective removal of P granule components phenolic glycolipid-1 (PGL-1) and PGL-3. Furthermore, mutating the methylated arginine residues on PGL-1 and PGL-3 resulted in impaired degradation of PGL-1 and PGL-3 [145]. These results indicate that modification of autophagic cargo proteins by arginine methylation may provide a regulatory mechanism for modulating autophagic degradation efficiency during selective autophagy. As discussed above, alcohol consumption impairs methionine metabolism and methylation reactions. It will be interesting to determine how these methylation changes would affect selective autophagy (such as mitophagy and lipophagy) and general autophagy in alcohol-induced liver disease in the future. The possible autophagic signaling pathways or targets modulated by ethanol are summarized in Figure 1.

## 5. Autophagy in Alcohol-Induced Pancreatitis

The pancreas is a glandular organ that has both endocrine and exocrine functions in vertebrates. In response to fasting or feeding, the pancreas secretes insulin or glucagon through its endocrine system (islet *β* or *α* cells) to maintain blood glucose levels. After a meal, the exocrine pancreas acinar cells release digestive enzymes into the pancreatic duct and ultimately the duodenal space. The exocrine acinar cells of the pancreas play a critical role in pancreatitis, and the hallmark changes of the acinar cells during pancreatitis include the intracellular activation of digestive enzymes, intracellular vacuolization, apoptosis, necrosis, edema, and inflammation [154, 155]. Acute and chronic pancreatitis are common gastrointestinal diseases that are potentially lethal with considerable morbidity and reduced life expectancy. Chronic pancreatitis is also associated with a high risk for the development of pancreatic adenocarcinoma, for which no treatment is currently available [156, 157].

While the molecular mechanisms for induction of acute pancreatitis (AP) are still poorly understood, several mechanisms have been identified that may play critical roles in the development of AP. Among them, premature activation of trypsin from trypsinogen within acinar cells to further trigger activation of the cascade of other pancreatic digestive zymogens has been considered a key pathogenic mechanism [154, 156]. This notion is also supported by the genetic evidence that mutations in cationic trypsinogen (*PRSS1*), pancreatic secretory trypsin inhibitor (*SPINK1*), and chymotrypsinogen C (*CTRC*) are associated with the susceptibility of pancreatitis [157]. However, the importance of the intra-acinar activation of trypsin has been challenged recently because it has been found that* trypsinogen isoform 7* (*T7*) KO mice have decreased trypsin activity but still develop pancreatitis upon cerulein-induced pancreatitis [158]. Furthermore, it has also been shown that intra-acinar trypsinogen activation leads to induction of acinar cell apoptosis resulting in the resolution of acute inflammation without causing chronic pancreatitis accompanied by fibrosis [159]. Therefore, other pathways may also be critical in the development of AP. Induction of inflammatory mediators is also a key feature in AP, and nuclear factor-*κ*B (NF-*κ*B) has been shown to play a critical role in AP. However, both activation and inhibition of NF-*κ*B have been shown to exacerbate acinar cell injury from experimental animal pancreatitis models, and clearly more studies are needed to further clarify the exact role of NF-*κ*B in the pathogenesis of AP [160–164]. In addition, acinar cells have an extensive network of ER to produce large amount of digestive enzymes, and thus acinar cells are more susceptible to ER stress. Mice with an acinar cell-specific deletion of X-box binding protein 1 (XBP-1), one of the three important unfolded protein response (UPR) proteins in response to ER stress, have extensive acinar cell apoptosis followed by pancreas regeneration [165]. Chronic ethanol feeding induces ER stress and the UPR response in mouse acinar cells and ethanol fed XBP1^+/−^ mice show loss of enzymogen granules and increased acinar cell death [166]. These findings suggest that ER stress and defective UPR may contribute to acinar cell death and pancreatitis. As an inflammatory disorder disease, acinar cell death is a key event in AP and both apoptotic and necrotic acinar cell death have been observed in experimental models of AP. It seems that acinar cell apoptosis can attenuate cerulein-induced acinar cell necrosis and protects against cerulein-induced AP by promoting caspase-mediated RIP cleavage [167]. Necrosis, which was initially thought of as a nonprogrammed cell death, has recently been shown to be highly regulated through formation of the necrosome. This programmed necrosis is also called necroptosis and is mainly mediated by RIP1–RIP3 signaling pathways. RIP1 and RIP3 are serine/threonine kinases, and their kinase activities are necessary for the formation of the necrosome, which further recruits downstream mixed lineage kinase domain-like protein (MLKL) and phosphoglycerate mutase family member 5 (PGAM5) [168–171]. PGAM5 is a mitochondrial phosphoglycerate mutase, which can dephosphorylate dynamin-related protein 1 (Drp1) resulting in Drp1 mitochondrial translocation and mitochondrial fragmentation to trigger necroptosis [171]. RIP3 can also directly phosphorylate MLKL to cause the translocation of MLKL to the plasma membrane and subsequent membrane rupture and necrosis [172–174].* RIP3* or* MLKL* KO mice are resistant to cerulein-induced AP, suggesting that necroptosis plays a critical role in animal experimental AP, but its relevance to human AP is not clear.

Although prolonged alcohol abuse is correlated with the clinical symptoms of a vast array of pancreatic diseases, alcohol alone does not cause severe pancreatic damage in human. Only a minority of subjects (~10%) who abuse alcohol develop clinical pancreatitis, indicating that other cofactors like environmental and genetic elements also contribute to disease development [175]. Cigarette smoking [175–177] and dietary habits [178] may be involved in the progress of alcoholic pancreatitis. Smoking accelerates the deterioration of pancreatitis because it significantly increases the risk of pancreatic calcifications [179]. While high fat and protein diets appear to exacerbate the course of chronic pancreatitis, saturated fatty acids and vitamins, especially vitamin E, may play a protective role against the detrimental effects on the pancreas caused by alcohol [180, 181]. Moreover, it seems that gender and ethnicity may also be factors for alcoholic pancreatitis; that is, men have a higher risk than women, and African Americans have a greater chance for development of chronic alcoholic pancreatitis than other ethnic groups.

There are several widely used rodent models for nonalcoholic pancreatitis. Reliable AP animal models should reproduce the clinical pathophysiology, symptomatology, and etiology, such as a significant activation of serum pancreatic enzymes, remarkable histological changes, and pancreatitis-associated complications [182]. Choline-deficient, ethionine-supplemented diet is a widely used model for inducing pancreatitis since a synergistic action of choline deficiency with the basic toxicity of ethionine on acinar cells leads to intraparenchymal activation of zymogens [183]. Young female mice fed with this diet developed acute hemorrhagic pancreatitis with massive fat necrosis throughout the peritoneal cavity. However, the nonselective effects of this model hamper its ability to study pancreatitis-induced multiply organ dysfunction syndromes as it can directly affect liver and brain. The intraperitoneal injection of L-arginine can induce acute necrotizing pancreatitis in rats and mice [184, 185]. This model has high specificity and flexibility in controlling the extent of pancreatic severity, which makes it suitable for studying extrapancreatic organ damage, but the precise mechanism is not fully understood. Accumulated evidence suggests that nitric oxide (NO) [186], oxygen free radicals [187, 188], and inflammatory mediators [189, 190] are all involved in the progression of the disease. Treatment with supramaximal cholecystokinin (CCK) or its analogue cerulein induces pancreatitis in rodents, which has been studied extensively since the pathological and histological presentation of this model is similar to the early phase of AP in human [191]. Furthermore, both CCK and cerulein can be used to initiate hyperstimulation-induced pancreatitis in primary cultured acinar cells, which makes it a valuable tool for studying the pathophysiology and mechanisms of secretagogue-induced pancreatitis [192]. In addition to these noninvasive models, a closed duodenal loop-induced pancreatitis model is utilized for studying duodenal reflux-induced AP [193], and the duct obstruction model is used to mimic gallstone obstruction-induced AP in the clinical setting [194, 195]. Moreover, duct infusion-induced pancreatitis is also used in combination with bile acids, like taurocholate or glycodeoxycholic acid to trigger necrotizing AP [196].

Similar to the animal models for alcohol-induced liver injury, neither acute nor chronic administration of ethanol alone in rodents leads to pancreatitis. Alcohol-induced pancreatitis requires other additional factors such as a viral infection, a high fat diet or submaximal postprandial dose of CCK or cerulein or cholinergic stimulation (such as by carbachol). Though the precise mechanisms by which alcohol induces pancreatic damage remain vague, several mechanisms have been suggested. A series of elegant published works from Gaisano's group showed that alcohol consumption may alter apical and basolateral exocytosis in pancreatic acinar cells [197, 198]. Mechanistically, it has been shown that alcohol induced protein kinase C*α*, which phosphorylated Munc18c and displaced it from binding to basolateral plasma membrane syntaxin 4 (Syn-4), which results in formation of the Syn4/synaptosomal-associated protein 23 (SNAP23)/vesicle-associated membrane protein 8 (VAMP8) fusion complex. The Syn4/SNAP23/VAMP8 fusion complex then redirected the zymogen from apical exocytosis to basolateral exocytosis, which causes pancreatitis [197–199]. Chronic ethanol feeding promotes a shift of acinar cell apoptosis to necrosis, but little is known about the mechanisms involved. In pancreas, ethanol is metabolized through both oxidative and nonoxidative pathways. Oxidative metabolism of ethanol is mediated by ADH in cytosol and ALDH2 in mitochondria, which generates acetaldehyde and acetate, respectively. Nonoxidative metabolism converts ethanol to FAEE via fatty acyl responsive regulator (FarR) synthase. It has been shown that oxidative metabolism of ethanol causes mitochondrial failure by activating mitochondrial permeability transition, a key event in regulating cell death [200]. However, whether ethanol feeding would affect other necrotic proteins such as RIP3 in pancreas is not known although RIP3 has been reported to be important in mediating ethanol feeding-induced liver injury in mice [48]. In addition, as discussed above, ethanol feeding may also induce ER stress in acinar cells to trigger cell death [166]. Alcohol consumption can increase gut permeability, which causes bacterial translocation across the mucosal barrier, and leads to the elevation of lipopolysaccharide (LPS) levels. Alcohol-fed rats that were further treated with LPS had increased expression of TGF-*β*, which led to subsequent pancreatic fibrosis [3].

Increasing evidence now supports the role of autophagy in both alcoholic and nonalcoholic pancreatitis, and it is generally thought that lysosomal/autophagic dysfunction can initiate pancreatitis. It has long been noted that there is an increased accumulation of large intracellular vacuoles in acinar cells in both experimental and human pancreatitis, and recent evidence indicates that the nature of these vacuoles is autophagic and lysosomal origin because these structures have double-membrane and are positive for LC3-II. In cerulein-induced acute AP, there was an increase in autophagosome numbers but autophagic flux was impaired due to lysosomal dysfunction [201]. Decreased Lamp-2 proteins and possible fusion of autophagosomes with lysosomes were also found in alcohol- and LPS-induced AP [202]. Moreover, XBP1^+/−^ mice fed with chronic ethanol had increased acinar cell death with loss of enzymogen granules. These mice also had increased LC3-II levels in acinar cells although autophagic flux assay was not performed in this study [166]. Mechanistically, it has been suggested that autophagy may help remove zymogens through a selective process termed zymophagy, which is regulated by the vacuole membrane protein- (VMP1-) USP9x-p62/SQSTM1 complex and attenuates intra-acinar trypsinogen activation. VMP1 interacts with Beclin-1 to promote the formation of autophagosomes, and it also interacts with the ubiquitin-protease USP9x to induce selective zymophagy, which prevents acinar cell death [203, 204]. In addition, results from Gukovskaya's group suggest that inefficient autophagic degradation of zymogens due to defective lysosomal proteolytic activity may promote pancreatitis. They also proposed that an imbalance between cathepsin B (CatB) and cathepsin L (CatL) may result in decreased degradation of trypsin, which leads to pancreatitis [155, 192]. Moreover, mice with pancreas-specific deletion of IKK-*α*, an essential component for NF-*κ*B activation, develop spontaneous pancreatitis. Interestingly, decreased autophagic flux has been found in the mouse pancreas with specific deletion of IKK-*α*. Similar to the autophagy-deficient liver, increased p62/SQSTM1 levels were also found in the IKK-*α*-deficient pancreas, and further deletion of p62/SQSTM1 in the pancreas attenuated pancreatitis in pancreas-specific IKK-*α*-deficient mice [160]. Taken together, it seems that all of the above evidence supports that impaired autophagy may contribute to pancreatitis. Nevertheless, an early study using pancreas acinar cell-specific-*Atg5* KO mice showed decreased acinar cell vacuolization and pancreatitis after cerulein treatment, and the authors proposed that autophagy machinery may be required for the trypsinogen activation to induce pancreatitis [205]. These results from acinar cell-specific* Atg5*-KO mice seem to be contradictory to the above other findings that suggest impaired autophagy promotes pancreatitis. However, Atg5 mainly regulates the upstream formation of autophagosomes, and it is possible that upstream autophagy (autophagosome biogenesis) and downstream of autophagy (autolysosome degradation) could play different roles in pancreatitis. Trypsinogen may use autophagosomes as vehicles for transport to lysosomes where trypsinogen is activated. Indeed, inhibition of the early phase of autophagy by 3-methyladenine (3-MA) completely blocked trypsinogen activation [201]. In contrast, impaired functions of downstream autolysosomes also led to trypsinogen activation and pancreatitis [155, 206]. Therefore, it is possible that targeting different phases of autophagy may lead to different outcomes of pancreatitis. Suppression of early phase autophagosome formation and improvement of late autolysosome functions may attenuate pancreatitis, but future experiments are needed to test this hypothesis.

## 6. Autophagy in Other Tissue Injury Induced by Alcohol

### 6.1. Heart

Heart is mainly comprised of long-lived and postmitotic cardiomyocytes. Increasing evidence indicates that autophagy plays an important role in maintaining the function and viability of cardiomyocytes by controlling the homeostasis of intracellular proteins, energy, and organelles [207]. Studies from genetic KO animal models, such as using the cardiomyocyte-specific* Atg5* KO mice, revealed that basal autophagy plays a vital housekeeping role in removing damaged organelles and proteins in cardiomyocytes to maintain their normal functions [208]. In contrast, both protective and detrimental roles of autophagy have been reported in “stressed” or “diseased” heart. For example, induction of autophagy is protective against ischemia-induced heart injury, whereas autophagy could be detrimental in pressure overload-induced heart failure and during reperfusion [209, 210].

Low to moderate alcohol consumption is beneficial to patients with cardiovascular events [211]. In contrast, heavy alcohol consumption impairs cardiac geometry and function [5] and increases the incidence of sudden cardiac death and ventricular arrhythmias [212]. Whether autophagy plays a protective or detrimental role in alcoholic heart disease is not fully understood. Jun Ren's team has conducted a series of studies on ethanol-induced cardiac dysfunction with a focus on autophagy [213–217]. In both binge [213] and chronic [214] alcohol models, heart LC3-II levels were increased in an AMPK-dependent manner. Furthermore results from this group's studies tend to suggest that autophagy may contribute to alcohol-induced malfunction of cardiomyocytes. Acute ethanol treatment led to compromised heart functions with decreased fractional shortening, peak shortening, and an intracellular Ca^2+^ rise in mouse cardiomyocytes. Acute ethanol exposure also increased LC3-II level, which was accompanied by increased phosphorylation of AMPK and Raptor and decreased phosphorylation of mTOR and ULK1 in mouse cardiomyocytes. Interestingly, pharmacological or genetic inhibition of AMPK attenuated ethanol-induced autophagosome formation and cardiomyocyte apoptosis. Moreover, 3-MA reversed ethanol-induced cardiomyocyte contractile defects [215]. Similar to acute ethanol treatment, chronic ethanol feeding also led to increased autophagosome formation in mouse cardiomyocytes with heart hypertrophy and cardiomyocyte contractile anomalies, and 3-MA treatment also ablated this ethanol-induced cardiomyocyte malfunction [216]. Moreover, mice with cardiac-specific overexpression of ADH, which metabolizes alcohol to acetaldehyde, were more susceptible to ethanol-induced autophagy changes and ethanol-induced damage of cardiomyocytes [216]. In contrast, transgenic mice overexpressing ALDH2, which converts acetaldehyde to acetate during alcohol metabolism, blunted chronic alcohol-induced mTOR inhibition and increased LC3-II levels resulting in improved cardiac geometry and function in alcohol-treated mice [214]. These findings suggest that the ethanol metabolite acetaldehyde may account for ethanol-mediated autophagy changes and impaired cardiac functions.

While these data generally suggest that either acute or chronic ethanol treatment may induce autophagy and contribute to ethanol-induced malfunction of cardiomyocytes, no clear autophagic flux data were shown in these studies. It is intriguing that acute ethanol treatment increased both LC3-II and p62/SQSTM1 in mouse cardiomyocytes [215], although it is not clear whether the increased p62/SQSTM1 was due to decreased degradation or increased transcription. More studies, in particular the use of genetic autophagy-deficient animal models, are definitely needed to further clarify the autophagy status after alcohol exposure and the exact role of autophagy in alcohol-induced heart dysfunction.

### 6.2. Brain

It is well established that excessive ethanol intake results in regional brain damage and cognitive dysfunction [6, 218]. Potential mechanisms that are responsible for alcohol-induced brain injury include higher sensitivity for excitotoxicity, impaired catabolism of homocysteine, reduced neurotrophic factors, failure to repair damaged DNA, acetaldehyde adduct formation, and so on [218]. It is generally thought that, in the mature mammalian brain, autophagy is hard to detect even under nutrient deprivation conditions [219]. This is probably due to the vital functions of brain that need to be protected from even systemic nutrient deprivation. However, neural cell-specific* Atg5* KO mice have increased accumulation of cytoplasmic inclusion bodies in neurons and develop progressive deficits in motor function, suggesting that basal autophagy in the brain is important for preventing the accumulation of abnormal proteins to preserve neural function and protects against neurodegeneration [220]. Moreover, both increased and impaired autophagy have also been observed in various acute brain injuries including those induced by alcohol [221, 222].

Ethanol treatment increased autophagic flux in SH-SY5Y neuroblastoma cells and in the developing mouse brain through inhibition of mTOR. More importantly, induction of autophagy by rapamycin attenuated ethanol-induced ROS production and neuronal cell death in SH-SY5Y cells and in the mouse developing brain. In contrast, inhibition of autophagy either by wortmannin or shRNA knockdown of Beclin-1 exacerbated ethanol-induced neurotoxicity [222]. Moreover, hypoxic preconditioning activated autophagy and protected against ethanol-induced neurotoxicity, which was abolished when autophagy was inhibited by either bafilomycin A 1 or wortmannin [223]. These results suggest that autophagy protects against ethanol-induced neuronal injury. Fetal alcohol spectrum disorder (FASD) results from prenatal exposure to alcohol, which is the leading cause of mental retardation. Children with FASD often have neuropsychological and behavioral problems and develop secondary disabilities including depression and anxiety disorder. Alimov et al. [224] found that subcutaneous injection with ethanol induced neuroapoptosis in postnatal day 4 mice but not in postnatal day 12 mice. Interestingly, they further found that the expression of genes that regulate autophagy and the UPR was lower whereas the expression of proapoptotic genes was higher in postnatal day 4 mice than postnatal day 12 mice. These results imply that decreased autophagy activity may contribute to the vulnerability of the immature brain to alcohol exposure. However, a more recent study found that administration with 10% (v/v) ethanol for 4 month led to an accumulation of polyubiquitinated proteins in the mouse cerebral cortex likely due to an impaired ubiquitin-proteasome system and autophagy. Specifically, it was found that ethanol treatment increased mTOR activity and decreased expression of several Atg genes including* Atg12*,* Atg5*,* p62/SQSTM1,* and* LC3*. Ethanol treatment also increased brain inflammatory mediators such as IFN-*γ*. Interestingly, these ethanol-induced changes were attenuated in* toll-like receptor 4* (*TLR4*) KO mice, which were protected against chronic ethanol exposure-induced brain injury [225]. These results suggest that ethanol-induced impairment of the ubiquitin proteasome system and autophagy could be due to the activation of TLR4 by inflammatory mediators. In the future, more studies are needed to determine whether autophagy is activated or impaired after alcohol consumption using different animal models such as Gao-binge model. Moreover, it will also be interesting to determine whether the metabolism of ethanol is required for ethanol-induced changes on autophagy in brain. Nevertheless, it seems that activation of autophagy is beneficial for alcohol-induced brain injury.

### 6.3. Muscle

Skeletal muscles are composed of myofibers that control our body's motion. Myofibers are composed of myofibrils that form highly organized units called sarcomeres, which contain repeated actin and myosin filaments. Approximately 40% of our body mass is from skeletal muscle, which also plays a critical role in regulating metabolism by providing amino acids through breaking proteins and organelles to meet the energy needs of the body [226]. Thus, it is not surprising that emerging evidence suggests that autophagy is important for controlling muscle mass. Modulating muscle autophagy also influences exercise and energy and lipid metabolism [226, 227]. Both beneficial and deleterious roles of autophagy in regulating muscle mass/wasting have been proposed. Activation of FoxO3 led to increased expression of Atg genes and activation of autophagy, which resulted in muscle atrophy [127, 128]. MTMR14 is a lipid phosphatase that antagonizes VPS34 to dephosphorylate PI3-P to phosphatidylinositol (PI) and thus inhibits autophagy. Increased autophagy and muscle atrophy have been reported in* MTMR14* knockdown zebrafish. Moreover, centronuclear myopathy was also found in humans that have* MTMR14* mutations [228]. Paradoxically, muscle-specific* Atg7* KO mice also developed myofiber degeneration and muscle atrophy accompanied with increased accumulation of protein aggregates, abnormal mitochondria, sarcoplasmic reticulum distension, vacuolization, increased oxidative stress, and apoptosis [229]. It is possible that autophagic degradation of proteins may lead to muscle atrophy whereas the muscle atrophy observed in the muscle autophagy-deficient mice is a maladaptive response due to the chronic loss of autophagy.

In addition to regulating muscle mass, autophagy in muscle also regulates body glucose and lipid metabolism. It has been shown that exercise induces autophagy in multiple organs involved in metabolic regulation including muscle, liver, pancreas, and adipose tissue [230]. Exercise increases Bcl-2 phosphorylation resulting in its dissociation from Beclin-1, which leads to the initiation of autophagy. Nonphosphorylatable mutation in Bcl-2 (Thr69Ala, Ser70Ala and Ser84Ala, Bcl2 AAA) knock-in mice causes them to be defective in exercise- and starvation-induced autophagy, and they show decreased exercise endurance. These defects are due to impaired exercise-induced skeletal muscle glucose uptake because of a loss in glucose transporter 4 (GLUT4) translocation [230, 231]. These findings suggest that autophagy may be beneficial for glucose homeostasis during exercise. Moreover, studies from muscle-specific* Atg7* KO mice also reveal that autophagy in muscle may regulate glucose and lipid homeostasis [232].* Atg7* muscle-specific KO mice have decreased fat mass and are resistant to high fat diet-induced obesity and insulin resistance. Mechanistically, it has been suggested that loss of* Atg7* may lead to accumulation of damaged mitochondria, which induces an Atf4-dependent production of fibroblast growth factor 21 (Fgf21) that increases fatty acid oxidation and browning of white adipose tissue (WAT) [232]. Thus, these seemingly beneficial effects of loss of muscle autophagy on glucose and lipid metabolism could be a secondary adaptive response in response to organelle damage induced by the loss of autophagy. It is not clear how long these adaptive responses would last and whether maladaptive responses would develop after long-term loss of muscle autophagy.

It is well known that chronic alcoholics have severe muscle loss and myopathy. Both* in vivo* and* in vitro* studies show that ethanol can inhibit skeletal muscle protein synthesis, which is likely mediated by increased expression of insulin-like growth factor binding protein-1 and myostatin (a TGF*β* superfamily member) resulting in the inhibition of mTOR and limitation of translational efficiency [7, 233, 234]. Using skeletal muscle biopsies from alcoholic cirrhotics, gastrocnemius from ethanol and pair-fed mice, and ethanol-exposed murine myotubes, Thapaliya et al. [235] provided evidence that autophagy contributes to alcohol-induced skeletal muscle loss. Using a standard CT imaging technique, it was found that alcoholic cirrhotics had lower muscle mass than controls. Interestingly, proteasome components and activity were decreased in alcoholic biopsy samples, suggesting that decreased skeletal mass in alcoholic cirrhotics is less likely mediated by the proteasome. Indeed, they found that the expression of several essential Atg genes and autophagic flux were increased in alcoholic biopsy samples, ethanol-fed mice, and ethanol-treated C2C12 murine myotubes. Alcohol-induced autophagy was mediated by acetaldehyde, the metabolite of ethanol, rather than ethanol* per se*. More importantly, pharmacological or genetic inhibition of autophagy mitigated the proteolysis of myotubes and the reduction of muscle mass [235]. However, most of the results were obtained from short term alcohol exposure experiments. It is uncertain whether long-term blockage of autophagy would be beneficial for alcohol-induced muscle loss. More studies are needed to further dissect the underlying mechanisms by which autophagy regulates skeletal muscle mass in alcoholics.

## 7. Concluding Remarks and Future Perspective

**Figure 2:**
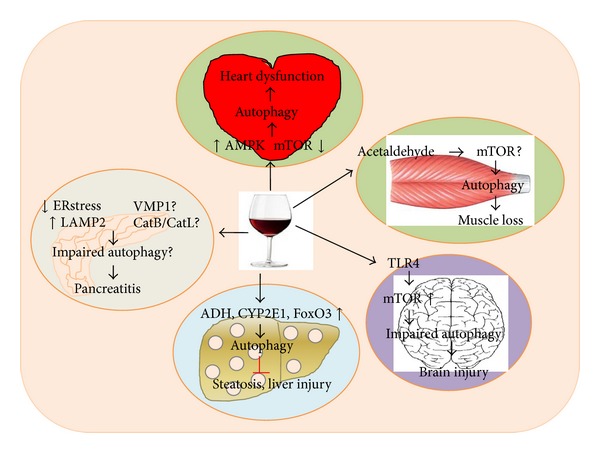
Differential roles of autophagy in alcohol-induced multitissue injury. Emerging evidence now indicates that alcohol consumption can either activate or impair autophagy as either a cellular adaptive/compensatory protective mechanism or as a detrimental factor contributing to alcohol-induced injury in various tissues/organs. In liver, it seems that alcohol metabolism through ADH and Cyp2E1 is required for autophagy activation. Acute alcohol treatment also induces FoxO3-mediated autophagy. Autophagy seems to selectively remove damaged mitochondria and excess lipid droplets and in turn attenuate alcohol-induced steatosis and liver injury. In pancreas, alcohol can induce ER stress and also decrease LAMP2 in the presence of endotoxin LPS, which leads to impaired autophagy resulting in pancreatitis. It is not known whether alcohol consumption would affect VMP1 and the ratio of CatB (cathepsin B)/CatL (cathepsin L), two important factors that regulate autophagy and pancreatitis, respectively. In heart, alcohol may activate autophagy through activating AMPK and inactivating mTOR. Autophagy activation seems to contribute to alcohol-induced heart dysfunction. In muscle, metabolism of alcohol to acetaldehyde activates autophagy resulting in muscle loss. Whether alcohol-induced autophagy in muscle is mediated by mTOR is not clear. In brain, alcohol increases mTOR and impairs autophagy in the mouse cerebral cortex resulting in brain injury, which is TLR4 dependent. Together, it is clear that alcohol can affect the autophagy process and in turn regulate tissue injury in various tissues/organs.

Recent rapid research progress has significantly enriched our knowledge on the molecular mechanisms regulating autophagy and its impact on human diseases. As outlined in this review, autophagy plays significant roles in alcohol consumption-induced multiple tissue/organ injuries including hepatic steatosis and liver injury, pancreatitis, impaired heart function, brain damage, and loss of muscle mass. While autophagy has been generally considered as a cell survival mechanism, both beneficial and detrimental effects of autophagy have been reported in alcohol-induced multiple tissue/organ injuries (Table 1). As a critical cellular mechanism sentinel for the homeostasis of proteins, energy, and organelles, autophagy may be beneficial for alcohol-induced liver injury through removing damaged mitochondria and lipid droplets, for AP through preventing zymogen activation and for brain injury through inhibiting ROS generation. However, autophagy seems to be detrimental for alcohol-induced heart malfunction and muscle atrophy, although more studies are needed to further confirm these concepts due to limited research and lack of clear autophagic flux data in these two areas (Figure 2). Given the dynamic nature of autophagy and the chronic alcohol consumption process, we are still facing great challenges to monitor the autophagy status* in vivo* for chronic diseases induced by alcohol consumption. Similarly, it is also difficult to monitor the autophagy status* in vivo* after chronic modulation of autophagy using either pharmacological autophagy inducers or inhibitors. More reliable* in vivo* autophagic flux assays are urgently needed to help further assess the therapeutic potential of pharmacological modulation of autophagy as a means to treat alcohol-induced tissue injuries.

## Figures and Tables

**Table 1:** Summary of *in vivo* studies on autophagy in alcohol-induced tissue injury.

	Model	Level of autophagy	Role of autophagy	References
Liver	Acute	Activated	Protective	Ding et al., 2010 [90]; Ni et al., 2013 [89]; Thomes et al., 2013 [93]
		Impaired∗	Protective	Wu et al., 2012 [91]; Yang et al., 2014 [100]; Zeng et al., 2012 [108]
	Chronic	Activated	Protective	Lin et al., 2013 [99]

Pancreas	Chronic	Impaired	Protective	Fortunato et al., 2009 [202]

Heart	Acute	Activated∗	Detrimental	Ge et al., 2011 [213]; Guo and Ren, 2012 [215]; Kandadi et al., 2013 [217]
	Chronic	Activated∗	Detrimental	Ge and Ren, 2012 [214]; Guo et al., 2012 [216]

Brain	Acute	Activated	Protective	Chen et al., 2012 [222], Alimov et al., 2013 [224]; Wang et al., 2013 [223]
	Chronic	Impaired	Protective	Pla et al., 2014 [225]

Skeletal muscle	Chronic	Activated	Detrimental	Thapaliya et al., 2014 [235]

Note: ∗autophagy flux assay is lacked.

## Abbreviations

ADH: Alcohol dehydrogenase
AICAR: 5-Amino-4-imidazole carboxamide riboside
Akt/PKB: Protein kinase B
ALD: Alcoholic liver disease
ALDH: Aldehyde dehydrogenase
Ambra1: Activating molecule in Beclin-1-regulated autophagy protein 1
AMPK: Amp-activated protein kinase
AP: Acute pancreatitis
Atg: Autophagy-related
Bcl-2: B-cell lymphoma 2
Bcl-xL: B-cell lymphoma-extra large
Bnip3: Bcl-2/adenovirus E1B 19 kDa interacting protein 3
CatB: Cathepsin B
CatL: Cathepsin L
CBS: Cystathionine b-synthase
CCK: Cholecystokinin
CP: Chronic pancreatitis
CTRC: Chymotrypsinogen C
Cyp2E1: Cytochrome P450, family 2, subfamily E, polypeptide 1
DFCP1: Double FYVE-containing protein 1
Drp1: Dynamin-related protein 1
EGFR: Epidermal growth factor receptor
ER: Endoplasmic reticulum
FarR: Fatty acyl responsive regulator
FIP200: FAK family-interacting protein of 200 kDa
FoxO: Forkhead box-containing protein, class O
gAcrp: Globular adiponectin
KO: Knockout
LAMP: Lysosomal-associated membrane protein
LC3: Microtubule-associated protein 1 light chain 3
LPS: Lipopolysaccharide
MLKL: Mixed lineage kinase domain-like protein
MTMR: Myotubularin-related protein
3-MA: 3-Methyladenine
MS: Methionine synthase
mTOR: Mammalian target of rapamycin
mTORC1: Mammalian target of rapamycin complex 1
NAD^+^/NADH: Nicotinamide adenine dinucleotide
NIAAA: National Institute on Alcohol Abuse and Alcoholism
NF-*κ*B: Nuclear factor-*Κ*b
Npr2: Natriuretic peptide receptor B
PGAM5: Phosphoglycerate mutase family member 5
PGL: Phenolic glycolipid
PI3K: Phosphoinositide 3-kinase
PI3P: Phosphatidylinositol 3-phosphate
PIP3: Phosphatidylinositol-3,4,5-trisphosphate
PP2A: Protein phosphatase 2A
Ppm1p: Protein phosphatase methyltransferase 1
PRMT: Protein arginine methyltransferases
PRSS1: Cationic trypsinogen
PTEN: Phosphatase and tensin homolog
RIP: Receptor interacting protein kinase
ROS: Reactive oxygen species
Rubicon: Run domain protein as Beclin-1 interacting and cysteine-rich containing
SAM: S-Adenosylmethionine
SIRT: Sirtuin
SNAP23: Synaptosomal-associated protein 23
SPINK1: Pancreatic secretory trypsin inhibitor
SQSTM1: Sequestosome 1 (p62)
SREBP-1: Sterol regulatory element-binding protein-1
STX17: Syntaxin 17
Syn-4: Syntaxin 4
T7: Trypsinogen isoform 7
TLR: Toll-like receptor
TOR: Target of rapamycin
TORC: Target of rapamycin complex
ULK1: Unc-51 like kinase 1
UPR: Unfolded protein response
UVRAG: UV irradiation resistance-associated gene
VAMP8: Vesicle-associated membrane protein 8
VMP1: Vacuole membrane protein 1
WIPI: WD-repeat domain phosphoinoside-interacting protein
XBP1: x-box binding protein 1.

## References

[1] Gao B, Bataller R (2011). Alcoholic liver disease: pathogenesis and new therapeutic targets. *Gastroenterology*.

[2] Gukovsky I, Lugea A, Shahsahebi M (2007). A rat model reproducing key pathological responses of alcoholic chronic pancreatitis. *The American Journal of Physiology—Gastrointestinal and Liver Physiology*.

[3] Gu H, Fortunato F, Bergmann F, Buchler MW, Whitcomb DC, Werner J (2013). Alcohol exacerbates LPS-induced fibrosis in subclinical acute pancreatitis. *American Journal of Pathology*.

[4] Piano MR, Phillips SA (2014). Alcoholic cardiomyopathy: pathophysiologic insights. *Cardiovascular Toxicology*.

[5] Ren J, Wold LE (2008). Mechanisms of alcoholic heart disease. *Therapeutic Advances in Cardiovascular Disease*.

[6] Harper C (2009). The neuropathology of alcohol-related brain damage. *Alcohol and Alcoholism*.

[7] Lang CH, Frost RA, Svanberg E, Vary TC (2004). IGF-I/IGFBP-3 ameliorates alterations in protein synthesis, eIF4E availability, and myostatin in alcohol-fed rats. *The American Journal of Physiology: Endocrinology and Metabolism*.

[8] Pruett D, Waterman EH, Caughey AB (2013). Fetal alcohol exposure: consequences, diagnosis, and treatment. *Obstetrical and Gynecological Survey*.

[9] Maurel DB, Boisseau N, Benhamou CL, Jaffre C (2012). Alcohol and bone: review of dose effects and mechanisms. *Osteoporosis International*.

[10] Molina PE, McClain C, Valla D (2002). Molecular pathology and clinical aspects of alcohol-induced tissue injury. *Alcoholism: Clinical and Experimental Research*.

[11] Xie Z, Klionsky DJ (2007). Autophagosome formation: core machinery and adaptations. *Nature Cell Biology*.

[12] Mizushima N, Levine B, Cuervo AM, Klionsky DJ (2008). Autophagy fights disease through cellular self-digestion. *Nature*.

[13] Ding WX, Manley S, Ni HM (2011). The emerging role of autophagy in alcoholic liver disease. *Experimental Biology and Medicine*.

[14] Dolganiuc A, Thomes PG, Ding W-X, Lemasters JJ, Donohue TM (2012). Autophagy in alcohol-induced liver diseases. *Alcoholism: Clinical and Experimental Research*.

[15] Czaja MJ, Ding WX, Donohue TM (2013). Functions of autophagy in normal and diseased liver. *Autophagy*.

[16] Crabb DW, Matsumoto M, Chang D, You M (2004). Overview of the role of alcohol dehydrogenase and aldehyde dehydrogenase and their variants in the genesis of alcohol-related pathology. *Proceedings of the Nutrition Society*.

[17] Chen CC, Lu RB, Chen YC (1999). Interaction between the functional polymorphisms of the alcohol-metabolism genes in protection against alcoholism. *American Journal of Human Genetics*.

[18] Lieber CS, DeCarli LM, Feinman L (1975). Effect of chronic alcohol consumption on ethanol and acetaldehyde metabolism. *Advances in Experimental Medicine and Biology*.

[19] Vonlaufen A, Wilson JS, Pirola RC, Apte MV (2007). Role of alcohol metabolism in chronic pancreatitis. *Alcohol Research and Health*.

[20] Zakhari S (2006). Overview: how is alcohol metabolized by the body?. *Alcohol Research & Health*.

[21] Lu Y, Cederbaum AI (2008). CYP2E1 and oxidative liver injury by alcohol. *Free Radical Biology and Medicine*.

[22] Zelner I, Matlow JN, Natekar A, Koren G (2013). Synthesis of fatty acid ethyl esters in mammalian tissues after ethanol exposure: a systematic review of the literature. *Drug Metabolism Reviews*.

[23] Wu H, Bhopale KK, Ansari GAS, Kaphalia BS (2008). Ethanol-induced cytotoxicity in rat pancreatic acinar AR42J cells: role of fatty acid ethyl esters. *Alcohol and Alcoholism*.

[24] Werner J, Saghir M, Warshaw AL (2002). Alcoholic pancreatitis in rats: injury from nonoxidative metabolites of ethanol. *American Journal of Physiology—Gastrointestinal and Liver Physiology*.

[25] Wu H, Cai P, Clemens DL, Jerrells TR, Ansari GA, Kaphalia BS (2006). Metabolic basis of ethanol-induced cytotoxicity in recombinant HepG2 cells: role of nonoxidative metabolism. *Toxicology and Applied Pharmacology*.

[26] Beckemeier ME, Bora PS (1998). Fatty acid ethyl esters: potentially toxic products of myocardial ethanol metabolism. *Journal of Molecular and Cellular Cardiology*.

[27] Bhopale KK, Wu H, Boor PJ, Popov VL, Ansari GAS, Kaphalia BS (2006). Metabolic basis of ethanol-induced hepatic and pancreatic injury in hepatic alcohol dehydrogenase deficient deer mice. *Alcohol*.

[28] Lange LG, Sobel BE (1983). Mitochondrial dysfunction induced by fatty acid ethyl esters, myocardial metabolites of ethanol. *The Journal of Clinical Investigation*.

[29] Haorah J, Knipe B, Gorantla S, Zheng J, Persidsky Y (2007). Alcohol-induced blood-brain barrier dysfunction is mediated via inositol 1,4,5-triphosphate receptor (IP3R)-gated intracellular calcium release. *Journal of Neurochemistry*.

[30] Zakhari S (2013). Alcohol metabolism and epigenetics changes. *Alcohol Research and Health*.

[31] Nanji AA, Hiller-Sturmhöfel S (1997). Apoptosis and necrosis: two types of cell death in alcoholic liver disease. *Alcohol Research and Health*.

[32] Ishii H, Adachi M, Fernández-Checa JC, Cederbaum AI, Deaciuc IV, Nanji AA (2003). Role of apoptosis in alcoholic liver injury. *Alcoholism: Clinical and Experimental Research*.

[33] Gukovskaya AS, Mareninova OA, Odinokova IV (2006). Cell death in pancreatitis: effects of alcohol. *Journal of Gastroenterology and Hepatology*.

[34] Wang Y, Hu R, Lugea A (2006). Ethanol feeding alters death signaling in the pancreas. *Pancreas*.

[35] Alfonso-Loeches S, Pascual-Lucas M, Blanco AM, Sanchez-Vera I, Guerri C (2010). Pivotal role of TLR4 receptors in alcohol-induced neuroinflammation and brain damage. *Journal of Neuroscience*.

[36] Johansson S, Ekström TJ, Marinova Z (2009). Dysregulation of cell death machinery in the prefrontal cortex of human alcoholics. *International Journal of Neuropsychopharmacology*.

[37] Olney JW, Wozniak DF, Jevtovic-Todorovic V, Farber NB, Bittigau P, Ikonomidou C (2002). Drug-induced apoptotic neurodegeneration in the developing brain. *Brain Pathology*.

[38] Fernández-Solá J, Nicolás JM, Fatjó F (2003). Evidence of apoptosis in chronic alcoholic skeletal myopathy. *Human Pathology*.

[39] Dey A, Cederbaum AI (2006). Alcohol and oxidative liver injury. *Hepatology*.

[40] Rouach H, Fataccioli V, Gentil M, French SW, Morimoto M, Nordmann R (1997). Effect of chronic ethanol feeding on lipid peroxidation and protein oxidation in relation to liver pathology. *Hepatology*.

[41] Li J, French B, Wu Y (2004). Liver hypoxia and lack of recovery after reperfusion at high blood alcohol levels in the intragastric feeding model of alcohol liver disease. *Experimental and Molecular Pathology*.

[42] French SW (2004). The role of hypoxia in the pathogenesis of alcoholic liver disease. *Hepatology Research*.

[43] Venugopal SK, Chen J, Zhang Y, Clemens D, Follenzi A, Zern MA (2007). Role of MAPK phosphatase-1 in sustained activation of JNK during ethanol-induced apoptosis in hepatocyte-like VL-17A cells. *Journal of Biological Chemistry*.

[44] Ji C, Kaplowitz N (2003). Betaine decreases hyperhomocysteinemia, endoplasmic reticulum stress, and liver injury in alcohol-fed mice. *Gastroenterology*.

[45] Donohue TM, McVicker DL, Kharbanda KK, Chaisson ML, Zetterman RK (1994). Ethanol administration alters the proteolytic activity of hepatic lysosomes. *Alcoholism: Clinical and Experimental Research*.

[46] Osna NA, Donohue TM (2007). Implication of altered proteasome function in alcoholic liver injury. *World Journal of Gastroenterology*.

[47] Bardag-Gorce F, van Leeuwen FW, Nguyen V (2002). The role of the ubiquitin-proteasome pathway in the formation of Mallory bodies. *Experimental and Molecular Pathology*.

[48] Roychowdhury S, Mcmullen MR, Pisano SG, Liu X, Nagy LE (2013). Absence of receptor interacting protein kinase 3 prevents ethanol-induced liver injury. *Hepatology*.

[49] Takamura A, Komatsu M, Hara T (2011). Autophagy-deficient mice develop multiple liver tumors. *Genes and Development*.

[50] Komatsu M, Kurokawa H, Waguri S (2010). The selective autophagy substrate p62 activates the stress responsive transcription factor Nrf2 through inactivation of Keap1. *Nature Cell Biology*.

[51] Singh R, Kaushik S, Wang Y (2009). Autophagy regulates lipid metabolism. *Nature*.

[52] Ni HM, Woolbright B, Williams J (2014). Nrf2 promotes the development of fibrosis and tumorigenesis in mice with defective hepatic autophagy. *Journal of Hepatology*.

[53] Williams JA, Manley S, Ding WX (2014). New advances in molecular mechanisms and emerging therapeutic targets in alcoholic liver diseases. *World Journal of Gastroenterology*.

[54] Mathews S, Xu M, Wang H, Bertola A, Gao B (2014). Animals models of gastrointestinal and liver diseases. Animal models of alcohol-induced liver disease: pathophysiology, translational relevance, and challenges. *The American Journal of Physiology-Gastrointestinal and Liver Physiology*.

[55] Wu D, Cederbaum AI (2008). Development and properties of HepG2 cells that constitutively express CYP2E1. *Methods in Molecular Biology*.

[56] Donohue TM, Osna NA, Clemens DL (2006). Recombinant Hep G2 cells that express alcohol dehydrogenase and cytochrome P450 2E1 as a model of ethanol-elicited cytotoxicity. *International Journal of Biochemistry and Cell Biology*.

[57] de Duve C, Wattiaux R (1966). Functions of lysosomes. *Annual Review of Physiology*.

[58] Tsukada M, Ohsumi Y (1993). Isolation and characterization of autophagy-defective mutants of Saccharomyces cerevisiae. *FEBS Letters*.

[59] Thumm M, Egner R, Koch B (1994). Isolation of autophagocytosis mutants of Saccharomyces cerevisiae. *The FEBS Letters*.

[60] Hosokawa N, Hara T, Kaizuka T (2009). Nutrient-dependent mTORCl association with the ULK1-Atg13-FIP200 complex required for autophagy. *Molecular Biology of the Cell*.

[61] Chang Y-Y, Neufeld TP (2009). An Atg1/Atg13 complex with multiple roles in TOR-mediated autophagy regulation. *Molecular Biology of the Cell*.

[62] Egan DF, Shackelford DB, Mihaylova MM (2011). Phosphorylation of ULK1 (hATG1) by AMP-activated protein kinase connects energy sensing to mitophagy. *Science*.

[63] Hamasaki M, Furuta N, Matsuda A (2013). Autophagosomes form at ER-mitochondria contact sites. *Nature*.

[64] Matsunaga K, Morita E, Saitoh T (2010). Autophagy requires endoplasmic reticulum targeting of the PI3-kinase complex via Atg14L. *The Journal of Cell Biology*.

[65] Axe EL, Walker SA, Manifava M (2008). Autophagosome formation from membrane compartments enriched in phosphatidylinositol 3-phosphate and dynamically connected to the endoplasmic reticulum. *Journal of Cell Biology*.

[66] Proikas-Cezanne T, Waddell S, Gaugel A, Frickey T, Lupas A, Nordheim A (2004). WIPI-1*α* (WIPI49), a member of the novel 7-bladed WIPI protein family, is aberrantly expressed in human cancer and is linked to starvation-induced autophagy. *Oncogene*.

[67] Polson HEJ, de Lartigue J, Rigden DJ (2010). Mammalian Atg18 (WIPI2) localizes to omegasome-anchored phagophores and positively regulates LC3 lipidation. *Autophagy*.

[68] Maria Fimia G, Stoykova A, Romagnoli A (2007). Ambra1 regulates autophagy and development of the nervous system. *Nature*.

[69] Liang C, Feng P, Ku B (2006). Autophagic and tumour suppressor activity of a novel Beclin1-binding protein UVRAG. *Nature Cell Biology*.

[70] Takahashi Y, Coppola D, Matsushita N (2007). Bif-1 interacts with Beclin 1 through UVRAG and regulates autophagy and tumorigenesis. *Nature Cell Biology*.

[71] Pattingre S, Tassa A, Qu X (2005). Bcl-2 antiapoptotic proteins inhibit Beclin 1-dependent autophagy. *Cell*.

[72] Zhong Y, Wang QJ, Li X (2009). Distinct regulation of autophagic activity by Atg14L and Rubicon associated with Beclin 1-phosphatidylinositol-3-kinase complex. *Nature Cell Biology*.

[73] Sun Q, Zhang J, Fan W (2011). The RUN domain of Rubicon is important for hVps34 binding, lipid kinase inhibition, and autophagy suppression. *The Journal of Biological Chemistry*.

[74] Ohsumi Y (2001). Molecular dissection of autophagy: Two ubiquitin-like systems. *Nature Reviews Molecular Cell Biology*.

[75] Mizushima N, Noda T, Yoshimori T (1998). A protein conjugation system essential for autophagy. *Nature*.

[76] Ichimura Y, Kirisako T, Takao T (2000). A ubiquitin-like system mediates protein lipidation. *Nature*.

[77] Young ARJ, Chan EYW, Hu XW (2006). Starvation and ULK1-dependent cycling of mammalian Atg9 between the TGN and endosomes. *Journal of Cell Science*.

[78] Itakura E, Mizushima N (2010). Characterization of autophagosome formation site by a hierarchical analysis of mammalian Atg proteins. *Autophagy*.

[79] Vergne I, Roberts E, Elmaoued RA (2009). Control of autophagy initiation by phosphoinositide 3-phosphatase jumpy. *The EMBO Journal*.

[80] Taguchi-Atarashi N, Hamasaki M, Matsunaga K (2010). Modulation of local Ptdins3P levels by the PI phosphatase MTMR3 regulates constitutive autophagy. *Traffic*.

[81] Itakura E, Kishi-Itakura C, Mizushima N (2012). The hairpin-type tail-anchored SNARE syntaxin 17 targets to autophagosomes for fusion with endosomes/lysosomes. *Cell*.

[82] Huynh KK, Eskelinen E, Scott CC, Malevanets A, Saftig P, Grinstein S (2007). LAMP proteins are required for fusion of lysosomes with phagosomes. *EMBO Journal*.

[83] Jäger S, Bucci C, Tanida I (2004). Role for Rab7 in maturation of late autophagic vacuoles. *Journal of Cell Science*.

[84] Tanida I, Sou Y, Ezaki J, Minematsu-Ikeguchi N, Ueno T, Kominami E (2004). HsAtg4B/HsApg4B/autophagin-1 cleaves the carboxyl termini of three human Atg8 homologues and delipidates microtubule-associated protein light chain 3- and GABA_A_ receptor-associated protein-phospholipid conjugates. *The Journal of Biological Chemistry*.

[85] Mizushima N, Ohsumi Y, Yoshimori T (2002). Autophagosome formation in mammalian cells. *Cell Structure and Function*.

[86] Klionsky DJ, Abdalla FC, Abeliovich H (2012). Guidelines for the use and interpretation of assays for monitoring autophagy. *Autophagy*.

[87] Ni H-M, Boggess N, Mcgill MR (2012). Liver-specific loss of Atg5 causes persistent activation of Nrf2 and protects against acetaminophen-induced liver injury. *Toxicological Sciences*.

[88] Sahani MH, Itakura E, Mizushima N (2014). Expression of the autophagy substrate SQSTM1/p62 is restored during prolonged starvation depending on transcriptional upregulation and autophagy-derived amino acids. *Autophagy*.

[89] Ni HM, Du K, You M, Ding WX (2013). Critical role of FoxO3a in alcohol-induced autophagy and hepatotoxicity. *American Journal of Pathology*.

[90] Ding W, Li M, Chen X (2010). Autophagy reduces acute ethanol-induced hepatotoxicity and steatosis in mice. *Gastroenterology*.

[91] Wu D, Wang X, Zhou R, Yang L, Cederbaum AI (2012). Alcohol steatosis and cytotoxicity: the role of cytochrome P4502E1 and autophagy. *Free Radical Biology and Medicine*.

[92] Wu D, Wang X, Zhou R, Cederbaum A (2010). CYP2E1 enhances ethanol-induced lipid accumulation but impairs autophaghy in HepG2 E47 cells. *Biochemical and Biophysical Research Communications*.

[93] Thomes PG, Ehlers RA, Trambly CS (2013). Multilevel regulation of autophagosome content by ethanol oxidation in HepG2 cells. *Autophagy*.

[94] Kharbanda KK, McVicker DL, Zetterman RK, MacDonald RG, Donohue TM (1997). Flow cytometric analysis of vesicular pH in rat hepatocytes after ethanol administration. *Hepatology*.

[95] Kharbanda KK, McVicker DL, Zetterman RK, Donohue TM (1995). Ethanol consumption reduces the proteolytic capacity and protease activities of hepatic lysosomes. *Biochimica et Biophysica Acta*.

[96] Donohue TM, Zetterman RK, Tuma DJ (1989). Effect of chronic ethanol administration on protein catabolism in rat liver. *Alcoholism: Clinical and Experimental Research*.

[97] Baraona E, Leo MA, Borowsky SA, Lieber CS (1975). Alcoholic hematomegaly: accumulation of protein in the liver. *Science*.

[98] Donohue TM (2009). Autophagy and ethanol-induced liver injury. *World Journal of Gastroenterology*.

[99] Lin CW, Zhang H, Li M (2013). Pharmacological promotion of autophagy alleviates steatosis and injury in alcoholic and non-alcoholic fatty liver conditions in mice. *Journal of Hepatology*.

[100] Yang L, Rozenfeld R, Wu D, Devi LA, Zhang Z, Cederbaum A (2014). Cannabidiol protects liver from binge alcohol-induced steatosis by mechanisms including inhibition of oxidative stress and increase in autophagy. *Free Radical Biology and Medicine*.

[101] Yang Z, Klionsky DJ (2010). Mammalian autophagy: core molecular machinery and signaling regulation. *Current Opinion in Cell Biology*.

[102] Facchinetti V, Ouyang W, Wei H (2008). The mammalian target of rapamycin complex 2 controls folding and stability of Akt and protein kinase C. *The EMBO Journal*.

[103] Ikenoue T, Inoki K, Yang Q, Zhou X, Guan K (2008). Essential function of TORC2 in PKC and Akt turn motif phosphorylation, maturation and signalling. *The EMBO Journal*.

[104] Yeon JE, Califano S, Xu J, Wands JR, de La Monte SM (2003). Potential role of PTEN phosphatase in ethanol-impaired survival signaling in the liver. *Hepatology*.

[105] He J, de La Monte S, Wands JR (2007). Acute ethanol exposure inhibits insulin signaling in the liver. *Hepatology*.

[106] Porstmann T, Santos CR, Griffiths B (2008). SREBP activity is regulated by mTORC1 and contributes to Akt-dependent cell growth. *Cell Metabolism*.

[107] Jegga AG, Schneider L, Ouyang X, Zhang J (2011). Systems biology of the autophagy-lysosomal pathway. *Autophagy*.

[108] Zeng T, Zhang C-L, Song F-Y (2012). PI3K/Akt pathway activation was involved in acute ethanol-induced fatty liver in mice. *Toxicology*.

[109] Kim J, Kundu M, Viollet B, Guan K (2011). AMPK and mTOR regulate autophagy through direct phosphorylation of Ulk1. *Nature Cell Biology*.

[110] Inoki K, Zhu T, Guan K-L (2003). TSC2 mediates cellular energy response to control cell growth and survival. *Cell*.

[111] Gwinn DM, Shackelford DB, Egan DF (2008). AMPK phosphorylation of raptor mediates a metabolic checkpoint. *Molecular Cell*.

[112] Egan DF, Shackelford DB, Mihaylova MM (2011). Phosphorylation of ULK1 (hATG1) by AMP—activated protein kinase connects energy sensing to mitophagy. *Science*.

[113] Kim J, Kim YC, Fang C (2013). Differential regulation of distinct Vps34 complexes by AMPK in nutrient stress and autophagy. *Cell*.

[114] Samari HR, Seglen PO (1998). Inhibition of hepatocytic autophagy by adenosine, aminoimidazole-4-carboxamide riboside, and N6-mercaptopurine riboside. Evidence for involvement of amp-activated protein kinase. *The Journal of Biological Chemistry*.

[115] Vucicevic L, Misirkic M, Janjetovic K (2011). Compound C induces protective autophagy in cancer cells through AMPK inhibition-independent blockade of Akt/mTOR pathway. *Autophagy*.

[116] Hu M, Wang F, Li X (2012). Regulation of hepatic lipin-1 by ethanol: role of AMP-activated protein kinase/sterol regulatory element-binding protein 1 signaling in mice. *Hepatology*.

[117] You M, Matsumoto M, Pacold CM, Cho WK, Crabb DW (2004). The role of AMP-activated protein kinase in the action of ethanol in the liver. *Gastroenterology*.

[118] Thomes PG, Trambly CS, Thiele GM (2012). Proteasome activity and autophagosome content in liver are reciprocally regulated by ethanol treatment. *Biochemical and Biophysical Research Communications*.

[119] Scherz-Shouval R, Shvets E, Fass E, Shorer H, Gil L, Elazar Z (2007). Reactive oxygen species are essential for autophagy and specifically regulate the activity of Atg4. *The EMBO Journal*.

[120] Wu D, Cederbaum AI (2013). Inhibition of autophagy promotes CYP2E1-dependent toxicity in HepG2 cells via elevated oxidative stress, mitochondria dysfunction and activation of p38 and JNK MAPK. *Redox Biology*.

[121] Huang H, Tindall DJ (2007). Dynamic FoxO transcription factors. *Journal of Cell Science*.

[122] Tikhanovich I, Kuravi S, Campbell RV (2014). Regulation of FOXO3 by phosphorylation and methylation in hepatitis C virus infection and alcohol exposure. *Hepatology*.

[123] Li H, Liang J, Castrillon DH, DePinho RA, Olson EN, Liu Z (2007). FoxO4 regulates tumor necrosis factor alpha-directed smooth muscle cell migration by activating matrix metalloproteinase 9 gene transcription. *Molecular and Cellular Biology*.

[124] Lin L, Hron JD, Peng SL (2004). Regulation of NF-*κ*B, Th activation, and autoinflammation by the forkhead transcription factor Foxo3a. *Immunity*.

[125] Brunet A, Sweeney LB, Sturgill JF (2004). Stress-dependent regulation of FOXO transcription factors by the SIRT1 deacetylase. *Science*.

[126] Xie Q, Hao Y, Tao L (2012). Lysine methylation of FOXO3 regulates oxidative stress-induced neuronal cell death. *EMBO Reports*.

[127] Mammucari C, Milan G, Romanello V (2007). FoxO3 controls autophagy in skeletal muscle in vivo. *Cell Metabolism*.

[128] Zhao J, Brault JJ, Schild A (2007). FoxO3 coordinately activates protein degradation by the autophagic/lysosomal and proteasomal pathways in atrophying muscle cells. *Cell Metabolism*.

[129] van der Vos KE, Eliasson P, Proikas-Cezanne T (2012). Modulation of glutamine metabolism by the PI(3)K-PKB-FOXO network regulates autophagy. *Nature Cell Biology*.

[130] Zhao Y, Yang J, Liao W (2010). Cytosolic FoxO1 is essential for the induction of autophagy and tumour suppressor activity. *Nature Cell Biology*.

[131] Nakagawa T, Guarente L (2011). Sirtuins at a glance. *Journal of Cell Science*.

[132] Haigis MC, Sinclair DA (2010). Mammalian sirtuins: biological insights and disease relevance. *Annual Review of Pathology: Mechanisms of Disease*.

[133] In HL, Cao L, Mostoslavsky R (2008). A role for the NAD-dependent deacetylase Sirt1 in the regulation of autophagy. *Proceedings of the National Academy of Sciences of the United States of America*.

[134] Kume S, Uzu T, Horiike K (2010). Calorie restriction enhances cell adaptation to hypoxia through Sirt1-dependent mitochondrial autophagy in mouse aged kidney. *Journal of Clinical Investigation*.

[135] Howitz KT, Bitterman KJ, Cohen HY (2003). Small molecule activators of sirtuins extend Saccharomyces cerevisiae lifespan. *Nature*.

[136] Nepal S, Park P (2013). Activation of autophagy by globular adiponectin attenuates ethanol-induced apoptosis in HepG2 cells: involvement of AMPK/FoxO3A axis. *Biochimica et Biophysica Acta - Molecular Cell Research*.

[137] Nepal S, Kim MJ, Lee ES (2014). Modulation of Atg5 expression by globular adiponectin contributes to autophagy flux and suppression of ethanol-induced cell death in liver cells. *Food and Chemical Toxicology*.

[138] Mandal P, Pritchard MT, Nagy LE (2010). Anti-inflammatory pathways and alcoholic liver disease: role of an adiponectin/interleukin-10/heme oxygenase-1 pathway. *World Journal of Gastroenterology*.

[139] Greer EL, Oskoui PR, Banko MR (2007). The energy sensor AMP-activated protein kinase directly regulates the mammalian FOXO3 transcription factor. *Journal of Biological Chemistry*.

[140] Kharbanda KK (2009). Alcoholic liver disease and methionine metabolism. *Seminars in Liver Disease*.

[141] Kharbanda KK (2013). Methionine metabolic pathway in alcoholic liver injury. *Current Opinion in Clinical Nutrition and Metabolic Care*.

[142] Halsted CH, Medici V (2012). Aberrant hepatic methionine metabolism and gene methylation in the pathogenesis and treatment of alcoholic steatohepatitis. *International Journal of Hepatology*.

[143] Sutter BM, Wu X, Laxman S, Tu BP (2013). Methionine inhibits autophagy and promotes growth by inducing the SAM-responsive methylation of PP2A. *Cell*.

[144] Artal-Martinez de Narvajas A, Gomez TS, Zhang JS (2013). Epigenetic regulation of autophagy by the methyltransferase G9a. *Molecular and Cellular Biology*.

[145] Li S, Yang P, Tian E, Zhang H (2013). Arginine methylation modulates autophagic degradation of PGL granules in *C. elegans*. *Molecular Cell*.

[146] Ding WX, Yin XM (2012). Mitophagy: mechanisms, pathophysiological roles, and analysis. *Biological Chemistry*.

[147] Kirkin V, Lamark T, Johansen T, Dikic I (2009). NBR1 cooperates with p62 in selective autophagy of ubiquitinated targets. *Autophagy*.

[148] Manley S, Williams JA, Ding WX (2013). Role of p62/SQSTM1 in liver physiology and pathogenesis. *Experimental Biology and Medicine*.

[149] Ding W-X, Ni H-M, Li M (2010). Nix is critical to two distinct phases of mitophagy, reactive oxygen species-mediated autophagy induction and Parkin-ubiquitin-p62-mediated mitochondrial priming. *The Journal of Biological Chemistry*.

[150] Matsumoto G, Wada K, Okuno M, Kurosawa M, Nukina N (2011). Serine 403 phosphorylation of p62/SQSTM1 regulates selective autophagic clearance of ubiquitinated proteins. *Molecular Cell*.

[151] Ichimura Y, Waguri S, Sou Y (2013). Phosphorylation of p62 activates the Keap1-Nrf2 pathway during selective autophagy. *Molecular Cell*.

[152] Aoki Y, Kanki T, Hirota Y (2011). Phosphorylation of serine 114 on Atg32 mediates mitophagy. *Molecular Biology of the Cell*.

[153] Wild P, Farhan H, McEwan DG (2011). Phosphorylation of the autophagy receptor optineurin restricts Salmonella growth. *Science*.

[154] Gaisano HY, Gorelick FS (2009). New insights into the mechanisms of pancreatitis. *Gastroenterology*.

[155] Gukovskaya AS, Gukovsky I (2012). Autophagy and pancreatitis. *American Journal of Physiology—Gastrointestinal and Liver Physiology*.

[156] Gukovsky I, Li N, Todoric J, Gukovskaya A, Karin M (2013). Inflammation, autophagy, and obesity: common features in the pathogenesis of pancreatitis and pancreatic cancer. *Gastroenterology*.

[157] Chen JM, Férec C (2009). Chronic pancreatitis: genetics and pathogenesis. *Annual Review of Genomics and Human Genetics*.

[158] Dawra R, Sah RP, Dudeja V (2011). Intra-acinar trypsinogen activation mediates early stages of pancreatic injury but not inflammation in mice with acute pancreatitis. *Gastroenterology*.

[159] Gaiser S, Daniluk J, Liu Y (2011). Intracellular activation of trypsinogen in transgenic mice induces acute but not chronic pancreatitis. *Gut*.

[160] Li N, Wu X, Holzer RG (2013). Loss of acinar cell IKK*α* triggers spontaneous pancreatitis in mice. *Journal of Clinical Investigation*.

[161] Baumann B, Wagner M, Aleksic T (2007). Constitutive IKK2 activation in acinar cells is sufficient to induce pancreatitis in vivo. *Journal of Clinical Investigation*.

[162] Algül H, Treiber M, Lesina M (2007). Pancreas-specific RelA/p65 truncation increases susceptibility of acini to inflammation-associated cell death following cerulein pancreatitis. *The Journal of Clinical Investigation*.

[163] Neuhöfer P, Liang S, Einwächter H (2013). Deletion of I*κ*B activates RelA to reduce acute pancreatitis in mice through up-regulation of Spi2A. *Gastroenterology*.

[164] Huang H, Liu Y, Daniluk J (2013). Activation of nuclear factor-*κ*B in acinar cells increases the severity of pancreatitis in mice. *Gastroenterology*.

[165] Hess DA, Humphrey SE, Ishibashi J (2011). Extensive pancreas regeneration following acinar-specific disruption of Xbp1 in mice. *Gastroenterology*.

[166] Lugea A, Tischler D, Nguyen J (2011). Adaptive unfolded protein response attenuates alcohol-induced pancreatic damage. *Gastroenterology*.

[167] Mareninova OA, Sung K, Hong P (2006). Cell death in pancreatitis: caspases protect from necrotizing pancreatitis. *Journal of Biological Chemistry*.

[168] Vandenabeele P, Declercq W, van Herreweghe F, Berghe TV (2010). The role of the kinases RIP1 and RIP3 in TNF-induced necrosis. *Science Signaling*.

[169] He S, Wang L, Miao L (2009). Receptor interacting protein kinase-3 determines cellular necrotic response to TNF-*α*. *Cell*.

[170] Sun L, Wang H, Wang Z (2012). Mixed lineage kinase domain-like protein mediates necrosis signaling downstream of RIP3 kinase. *Cell*.

[171] Wang Z, Jiang H, Chen S, Du F, Wang X (2012). The mitochondrial phosphatase PGAM5 functions at the convergence point of multiple necrotic death pathways. *Cell*.

[172] Wang H, Sun L, Su L (2014). Mixed lineage kinase domain-like protein MLKL causes necrotic membrane disruption upon phosphorylation by RIP3. *Molecular Cell*.

[173] Chen X, Li W, Ren J (2014). Translocation of mixed lineage kinase domain-like protein to plasma membrane leads to necrotic cell death. *Cell Research*.

[174] Cai Z, Jitkaew S, Zhao J (2014). Plasma membrane translocation of trimerized MLKL protein is required for TNF-induced necroptosis. *Nature Cell Biology*.

[175] Dufour MC, Adamson MD (2003). The epidemiology of alcohol-induced pancreatitis. *Pancreas*.

[176] Yadav D, Whitcomb DC (2010). The role of alcohol and smoking in pancreatitis. *Nature Reviews Gastroenterology and Hepatology*.

[177] Talamini G, Bassi C, Falconi M (1999). Alcohol and smoking as risk factors in chronic pancreatitis and pancreatic cancer. *Digestive Diseases and Sciences*.

[178] Olsen GW, Mandel JS, Gibson RW, Wattenberg LW, Schuman LM (1989). A case-control study of pancreatic cancer and cigarettes, alcohol, coffee and diet. *The American Journal of Public Health*.

[179] Maisonneuve P, Lowenfels AB, Müllhaupt B (2005). Cigarette smoking accelerates progression of alcoholic chronic pancreatitis. *Gut*.

[180] Lankisch MR, Imoto M, Layer P, DiMagno EP (2001). The effect of small amounts of alcohol on the clinical course of chronic pancreatitis. *Mayo Clinic Proceedings*.

[181] Lin Y, Tamakoshi A, Hayakawa T, Ogawa M, Ohno Y (2001). Associations of alcohol drinking and nutrient intake with chronic pancreatitis: Findings from a case-control study in Japan. *American Journal of Gastroenterology*.

[182] Chan YC, Leung PS (2007). Acute pancreatitis: Animal models and recent advances in basic research. *Pancreas*.

[183] Lombardi B, Estes LW, Longnecker DS (1975). Acute hemorrhagic pancreatitis (massive necrosis) with fat necrosis induced in mice by DL ethionine fed with a choline deficient diet. *The American Journal of Pathology*.

[184] Tani S, Itoh H, Okabayashi Y (1990). New model of acute necrotizing pancreatitis induced by excessive doses of arginine in rats. *Digestive Diseases and Sciences*.

[185] Su KH, Cuthbertson C, Christophi C (2006). Review of experimental animal models of acute pancreatitis. *HPB*.

[186] Takács T, Czakó L, Morschl É (2002). The role of nitric oxide in edema formation in L-arginine-induced acute pancreatitis. *Pancreas*.

[187] Czako L, Takacs T, Varga IS (2000). Oxidative stress in distant organs and the effects of allopurinol during experimental acute pancreatitis. *International Journal of Pancreatology*.

[188] Czakó L, Takács T, Varga IS (2000). The pathogenesis of L-arginine-induced acute necrotizing pancreatitis: inflammatory mediators and endogenous cholecystokinin. *Journal of Physiology Paris*.

[189] Rakonczay Z, Jármay K, Kaszaki J (2003). NF-*κ*B activation is detrimental in arginine-induced acute pancreatitis. *Free Radical Biology & Medicine*.

[190] Takács T, Rakonczay Z, Varga IS (2002). Comparative effects of water immersion pretreatment on three different acute pancreatitis models in rats. *Biochemistry and Cell Biology*.

[191] Dabrowski A, Konturek SJ, Konturek JW, Gabryelewicz A (1999). Role of oxidative stress in the pathogenesis of caerulein-induced acute pancreatitis. *European Journal of Pharmacology*.

[192] Mareninova OA, Hermann K, French SW (2009). Impaired autophagic flux mediates acinar cell vacuole formation and trypsinogen activation in rodent models of acute pancreatitis. *Journal of Clinical Investigation*.

[193] Pfeffer RB, Stasior O, Hinton JW (1957). The clinical picture of the sequential development of acute hemorrhagic pancreatitis in the dog. *Surgical Forum*.

[194] Ohshio G, Saluja A, Steer ML (1991). Effects of short-term pancreatic duct obstruction in rats. *Gastroenterology*.

[195] Lerch MM, Saluja AK, Rünzi M, Dawra R, Saluja M, Steer ML (1993). Pancreatic duct obstruction triggers acute necrotizing pancreatitis in the opossum. *Gastroenterology*.

[196] Schmidt J, Rattner DW, Lewandrowski K (1992). A better model of acute pancreatitis for evaluating therapy. *Annals of Surgery*.

[197] Cosen-Binker LI, Binker MG, Wang CC, Hong W, Gaisano HY (2008). VAMP8 is the v-SNARE that mediates basolateral exocytosis in a mouse model of alcoholic pancreatitis. *The Journal of Clinical Investigation*.

[198] Cosen-Binker LI, Lam PPL, Binker MG, Gaisano HY (2007). Alcohol-induced protein kinase C*α* phosphorylation of Munc18c in carbachol-stimulated acini causes basolateral exocytosis. *Gastroenterology*.

[199] Lam PPL, Cosen Binker LI, Lugea A, Pandol SJ, Gaisano HY (2007). Alcohol redirects CCK-mediated apical exocytosis to the acinar basolateral membrane in alcoholic pancreatitis. *Traffic*.

[200] Shalbueva N, Mareninova OA, Gerloff A (2013). Effects of oxidative alcohol metabolism on the mitochondrial permeability transition pore and necrosis in a mouse model of alcoholic pancreatitis. *Gastroenterology*.

[201] Gukovsky I, Gukovskaya AS (2010). Impaired autophagy underlies key pathological responses of acute pancreatitis. *Autophagy*.

[202] Fortunato F, Bürgers H, Bergmann F (2009). Impaired autolysosome formation correlates with Lamp-2 depletion: role of apoptosis, autophagy, and necrosis in pancreatitis. *Gastroenterology*.

[203] Grasso D, Ropolo A, Lo Ré A (2011). Zymophagy, a novel selective autophagy pathway mediated by VMP1-USP9x-p62, prevents pancreatic cell death. *Journal of Biological Chemistry*.

[204] Ropolo A, Grasso D, Pardo R (2007). The pancreatitis-induced vacuole membrane protein 1 triggers autophagy in mammalian cells. *Journal of Biological Chemistry*.

[205] Hashimoto D, Ohmuraya M, Hirota M (2008). Involvement of autophagy in trypsinogen activation within the pancreatic acinar cells. *Journal of Cell Biology*.

[206] Gukovsky I, Pandol SJ, Mareninova OA, Shalbueva N, Jia W, Gukovskaya AS (2012). Impaired autophagy and organellar dysfunction in pancreatitis. *Journal of Gastroenterology and Hepatology*.

[207] Lavandero S, Troncoso R, Rothermel BA, Martinet W, Sadoshima J, Hill JA (2013). Cardiovascular autophagy: concepts, controversies, and perspectives. *Autophagy*.

[208] Nakai A, Yamaguchi O, Takeda T (2007). The role of autophagy in cardiomyocytes in the basal state and in response to hemodynamic stress. *Nature Medicine*.

[209] Zhu H, Tannous P, Johnstone JL (2007). Cardiac autophagy is a maladaptive response to hemodynamic stress. *The Journal of Clinical Investigation*.

[210] Matsui Y, Takagi H, Qu X (2007). Distinct roles of autophagy in the heart during ischemia and reperfusion: roles of AMP-activated protein kinase and beclin 1 in mediating autophagy. *Circulation Research*.

[211] Costanzo S, di Castelnuovo A, Donati MB, Iacoviello L, de Gaetano G (2010). Alcohol consumption and mortality in patients with cardiovascular disease. A meta-analysis. *Journal of the American College of Cardiology*.

[212] Spies CD, Sander M, Stangl K (2001). Effects of alcohol on the heart. *Current Opinion in Critical Care*.

[213] Ge W, Guo R, Ren J (2011). AMP-dependent kinase and autophagic flux are involved in aldehyde dehydrogenase-2-induced protection against cardiac toxicity of ethanol. *Free Radical Biology and Medicine*.

[214] Ge W, Ren J (2012). MTOR-STAT3-notch signalling contributes to ALDH2-induced protection against cardiac contractile dysfunction and autophagy under alcoholism. *Journal of Cellular and Molecular Medicine*.

[215] Guo R, Ren J (2012). Deficiency in AMPK attenuates ethanol-induced cardiac contractile dysfunction through inhibition of autophagosome formation. *Cardiovascular Research*.

[216] Guo R, Hu N, Kandadi MR, Ren J (2012). Facilitated ethanol metabolism promotes cardiomyocyte contractile dysfunction through autophagy in murine hearts. *Autophagy*.

[217] Kandadi MR, Hu N, Ren J (2013). ULK1 plays a critical role in AMPK-mediated myocardial autophagy and contractile dysfunction following acute alcohol challenge. *Current Pharmaceutical Design*.

[218] Harper C, Matsumoto I (2005). Ethanol and brain damage. *Current Opinion in Pharmacology*.

[219] Mizushima N, Yamamoto A, Matsui M, Yoshimori T, Ohsumi Y (2004). In vivo analysis of autophagy in response to nutrient starvation using transgenic mice expressing a fluorescent autophagosome marker. *Molecular Biology of the Cell*.

[220] Hara T, Nakamura K, Matsui M (2006). Suppression of basal autophagy in neural cells causes neurodegenerative disease in mice. *Nature*.

[221] Smith CM, Chen Y, Sullivan ML, Kochanek PM, Clark RSB (2011). Autophagy in acute brain injury: feast, famine, or folly?. *Neurobiology of Disease*.

[222] Chen G, Ke Z, Xu M (2012). Autophagy is a protective response to ethanol neurotoxicity. *Autophagy*.

[223] Wang H, Bower KA, Frank JA, Xu M, Luo J (2013). Hypoxic preconditioning alleviates ethanol neurotoxicity: the involvement of autophagy. *Neurotoxicity Research*.

[224] Alimov A, Wang H, Liu M (2013). Expression of autophagy and UPR genes in the developing brain during ethanol-sensitive and resistant periods. *Metabolic Brain Disease*.

[225] Pla A, Pascual M, Renau-Piqueras J, Guerri C (2014). TLR4 mediates the impairment of ubiquitin-proteasome and autophagy-lysosome pathways induced by ethanol treatment in brain. *Cell Death and Disease*.

[226] Sandri M (2010). Autophagy in skeletal muscle. *FEBS Letters*.

[227] Neel BA, Lin Y, Pessin JE (2013). Skeletal muscle autophagy: a new metabolic regulator. *Trends in Endocrinology & Metabolism*.

[228] Tosch V, Rohde HM, Tronchère H (2006). A novel PtdIns3P and PtdIns(3,5)P2 phosphatase with an inactivating variant in centronuclear myopathy. *Human Molecular Genetics*.

[229] Masiero E, Sandri M (2010). Autophagy inhibition induces atrophy and myopathy in adult skeletal muscles. *Autophagy*.

[230] He C, Bassik MC, Moresi V (2012). Exercise-induced BCL2-regulated autophagy is required for muscle glucose homeostasis. *Nature*.

[231] He C, Sumpter R, Levine B (2012). Exercise induces autophagy in peripheral tissues and in the brain. *Autophagy*.

[232] Kim KH, Jeong YT, Oh H (2013). Autophagy deficiency leads to protection from obesity and insulin resistance by inducing Fgf21 as a mitokine. *Nature Medicine*.

[233] Preedy VR, Macallan DC, Griffin GE, Cook EB, Palmer TN, Peters TJ (1997). Total contractile protein contents and gene expression in skeletal muscle in response to chronic ethanol consumption in the rat. *Alcohol*.

[234] Lang CH, Fan J, Lipton BP, Potter BJ, McDonough KH (1998). Modulation of the insulin-like growth factor system by chronic alcohol feeding. *Alcoholism: Clinical and Experimental Research*.

[235] Thapaliya S, Runkana A, McMullen MR (2014). Alcohol-induced autophagy contributes to loss in skeletal muscle mass. *Autophagy*.

